# Narya, a RING finger domain-containing protein, is required for meiotic DNA double-strand break formation and crossover maturation in *Drosophila melanogaster*

**DOI:** 10.1371/journal.pgen.1007886

**Published:** 2019-01-07

**Authors:** Cathleen M. Lake, Rachel J. Nielsen, Amanda M. Bonner, Salam Eche, Sanese White-Brown, Kim S. McKim, R. Scott Hawley

**Affiliations:** 1 Stowers Institute for Medical Research, Kansas City, Missouri, United States of America; 2 Waksman Institute and Department of Genetics, Rutgers, the State University of New Jersey, Piscataway, New Jersey, United States of America; 3 Department of Molecular and Integrative Physiology, University of Kansas Medical Center, Kansas City, Kansas, United States of America; University of California Davis, UNITED STATES

## Abstract

Meiotic recombination, which is necessary to ensure that homologous chromosomes segregate properly, begins with the induction of meiotic DNA double-strand breaks (DSBs) and ends with the repair of a subset of those breaks into crossovers. Here we investigate the roles of two paralogous genes, *CG12200* and *CG31053*, which we have named Narya and Nenya, respectively, due to their relationship with a structurally similar protein named Vilya. We find that *narya* recently evolved from *nenya* by a gene duplication event, and we show that these two RING finger domain-containing proteins are functionally redundant with respect to a critical role in DSB formation. Narya colocalizes with Vilya foci, which are known to define recombination nodules, or sites of crossover formation. A separation-of-function allele of *narya* retains the capacity for DSB formation but cannot mature those DSBs into crossovers. We further provide data on the physical interaction of Narya, Nenya and Vilya, as assayed by the yeast two-hybrid system. Together these data support the view that all three RING finger domain-containing proteins function in the formation of meiotic DNA DSBs and in the process of crossing over.

## Author summary

Errors in chromosome segregation during meiosis are the leading cause of miscarriages and can result in genetic abnormalities like Down syndrome or Turner syndrome. For chromosomes to segregate faithfully, they must recombine with their homolog during the early steps of meiosis. An essential component of the process of meiotic recombination is creating the lesions (double-strand breaks, DSBs) that are required to form a crossover with the homologous chromosome. Crossovers are required to ensure chromosomes segregate properly at the first meiotic division. In this study we have identified two genes, *narya* and *nenya*, that are essential in DSB formation. We found that *narya* arose from a duplication of *nenya*, and these two genes are functionally redundant. In addition to its role in DSB formation, *narya* also plays a role in processing DSBs into crossovers. Strengthening our knowledge about the mechanism by which Narya both creates DSBs and processes them into crossovers will lead to a better understanding of the process of meiotic chromosome segregation not only in flies but many other organisms, as these genes have homologs in yeast, worms, plants, mice and humans.

## Introduction

Homologous recombination is an essential feature of meiosis and is required to ensure proper chromosome segregation. Although several core aspects of meiosis are highly conserved, many of the proteins and structures that mediate meiosis have features that are unique to each model organism. This is most apparent when comparing the process of meiotic recombination in *Drosophila* to other model organisms.

Meiotic recombination begins with the induction of programmed DNA double-strand breaks (DSBs). In *Drosophila* (as well as *Caenorhabditis elegans*) this event occurs in the context of full-length synaptonemal complex (SC) [1,2,3]. Therefore, in flies, synapsis is not dependent on DSB formation, as it is in other model organisms like budding yeast, plants and mammals [4,5,6,7,8]. DSBs are catalyzed by the evolutionarily conserved protein Spo11 (MEI-W68 in *Drosophila* [9]), the homolog of subunit A of TopoVI DNA topoisomerase [10,11]. Although nine other DSB accessory proteins (Mre11, Rad50, Xrs2, Ski8, Rec102, Rec104, Rec114, Mei4 and Mer2) have been identified in budding yeast (reviewed in [12,13]), only three proteins have been demonstrated to be required for DSB formation in *Drosophila* (MEI-P22, Trem, and Vilya) [14,15,16]. MEI-P22 has sequence homology to the transducer domain found within the B subunit of TopoVI DNA topoisomerase [17], and therefore may interact directly with MEI-W68 as a complex. Trem is a C2H2 zinc finger domain protein with no known homologs in other model systems [15]. Vilya, a RING finger domain-containing protein, has homology to Zip3-like family members found in several organisms [16]. However, none of the members in other systems appear to affect the formation of DSBs themselves [18,19,20,21,22,23,24,25].

Once DSBs are made, they must be repaired into either crossovers or noncrossovers. This is a multistep process utilizing enzymes and proteins that stabilize crossover intermediates and further promote crossover maturation. Early-acting pro-crossover proteins in most organisms (yeast, plants, nematodes and mammals) include the heterodimer of Msh4 and Msh5 (reviewed in [26]). The Msh4/5 complex is required for stabilizing crossover intermediates and promoting repair through the crossover pathway. *Drosophila* lacks this complex and instead is thought to use the MEI-MCM complex (REC, MEI-217 and MEI-218) for this function [27,28,29]. In addition to the lack of conservation in early pro-crossover proteins, *Drosophila* also seems to lack the homologs of late pro-crossover proteins that are required for crossover maturation [29]. Instead of the endonuclease MutLγ (Mlh1 and Mlh3) that is used to resolve crossovers in most organisms, *Drosophila* appears to use an endonuclease complex consisting of MEI-9, Ercc1, Mus312 and Hdm (reviewed in [29,30]).

Although many of the yeast proteins necessary to create DSBs and determine their fate as crossovers or noncrossovers are not conserved in flies, we recently identified a protein named Vilya, which is required for DSB formation and localizes to the recombination nodule (RN), a protein structure assembled only at sites of crossing over [16]. Vilya appears to be homologous to the Zip3-like protein family that is involved in crossover fate by stabilizing crossover intermediates and aiding crossover maturation. In fact, Vilya appears to link DSB formation and crossover formation in *Drosophila*. Zip3-like proteins fall into two subgroups: the Zip3-RNF212 group and the HEI10 group, with all members of both groups sharing conserved structural properties (reviewed in [31]). Most of these Zip3-like proteins appear to have dynamic localization patterns that involve either a redistribution of the protein from the SC to sites of recombination intermediates and/or an increase in their concentration at these sites as recombination intermediates are processed into crossovers. Studies in multiple organisms argue Zip3-like proteins act as post-translational regulators at sites of crossing over either through sumoylation or ubiquitination or both [20,22,32,33,34,35,36,37]. Recently, a study in *C*. *elegans* identified three paralogs of a previously known member of this group, ZHP-3, which were shown to function in two separate heterodimeric complexes [25]. These complexes are thought to form a signaling network that mediates crossover assurance and crossover interference by functioning both to stabilize crossover intermediates (ZHP3/4) [25,38] and to promote crossover maturation (ZHP1/2) [25], similar to the roles found in mammalian RNF212 and HEI10, respectively.

Here we report on the identification of two paralogs, *narya* (*CG12200*) and *nenya* (*CG31053*), that encode proteins that are both structurally and functionally related to Vilya and have homology to the Zip3-like family. In *D*. *melanogaster*, *narya* likely arose from a gene duplication of *nenya* less than 40 million years ago, and the two show genetic redundancy and are required for meiotic DSB formation. Using the CRISPR-Cas9 system to tag the endogenous copy of *narya*, we find that Narya localizes to DSBs and colocalizes with Vilya throughout pachytene. As we previously showed Vilya to be a component of the RN, this would suggest that Narya (and likely Nenya as well) are also RN components. In addition, as is true for Vilya, the localization of Narya to discrete foci within the SC is dependent on DSB formation, and in the absence of DSBs, Narya localizes uniformly along the SC. Finally, we report the identification of a separation-of-function allele of *narya* (*narya*^*G4*^) that links Narya directly to crossover maturation. Therefore, Narya, and most likely Nenya, appear to be the second and third examples after Vilya of proteins linking DSB formation with DSB fate, and likely Narya is the second protein to make up the RN in *Drosophila*.

## Results

### Identification of *narya* and *nenya* across the *Drosophila* genus

Because many organisms have multiple Zip3-like proteins that play a role in meiosis, we conducted a genome-wide search for Zip3-related genes in *Drosophila melanogaster*. We identified two genes (*CG12200* and *CG31053*) that appeared to encode good Zip3-like candidates. *CG12200* (FBgn0031018) is located on the *X* chromosome at map position 18C7 in the last (6^th^) intron of *CG32533*. *CG32533* is a gene with unknown function that is predicted to be a helicase. *CG31053* (FBgn0051053) is located on the *3*^rd^ chromosome at map position 98B6 in the first intron of *minotaur* (*CG5508*), a conserved member of the glycerol-3-phosphate O-acyltransferase (GPAT) family. Both *CG12200* and *CG31053* are predicted to encode proteins that have similar structural properties to Zip3-like family members (including Vilya in *Drosophila* [16]), such as an N-terminal C3HC4 RING finger domain and an internal predicted coiled-coil domain (S1A Fig). Therefore, we named these genes *narya* (*CG12200*) and *nenya* (*CG31053*) to complete the Three Rings of Power given by the Elves of Eregion [39].

We used the protein sequences of Narya and Nenya to identify homologous proteins in other model organisms to determine if we could identify either Zip3-like family members or proteins outside of this family that had known roles specifically in meiosis or meiotic recombination. In addition to showing protein homology to Zip3 in budding yeast, Narya, Nenya and Vilya showed homology to all four Zip3-like family members in *C*. *elegans* (ZHP-1, ZHP-2, ZHP-3 and ZHP-4) and to RNF212 and RNF212B in several mammalian species. (RNF212B is a protein known to affect the recombination rate in both cattle and sheep [40,41].) All three of the *D*. *melanogaster* RING proteins (Narya, Nenya and Vilya) cluster with the Zip3-RNF212 subgroup (S1B Fig) [16].

**Fig 1:**
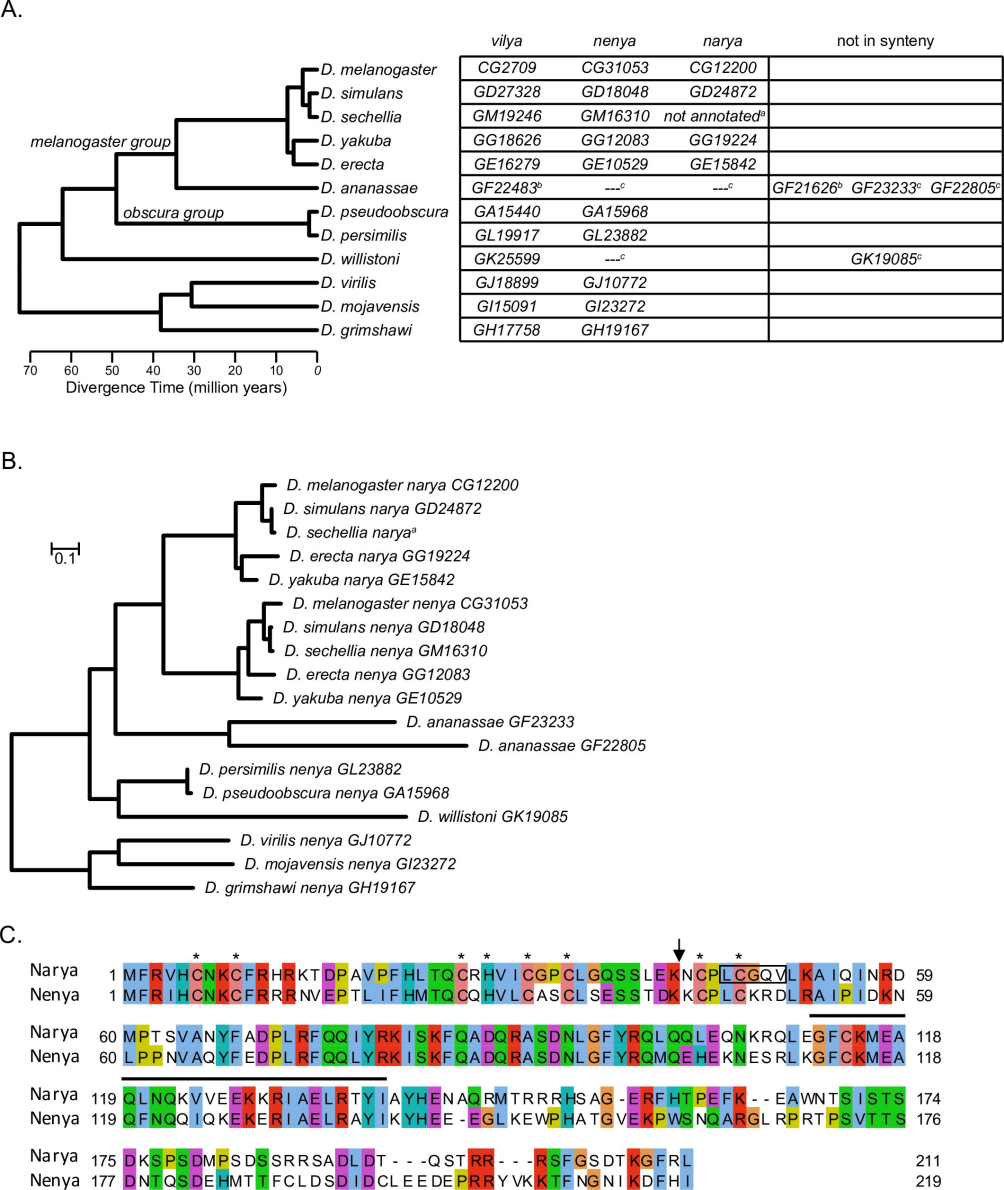
*narya* and *nenya* likely diverged less than 40 Mya. (A) Phylogenetic tree is shown to the left of the gene identifier for the homologs of *vilya*, *nenya* and *narya* in the 12 sequenced *Drosophila* species. Based on the gene location within the chromosome, and more specifically within the intron of the homolog of *CG32533* (for *narya*) and *minotaur* (for *nenya*) in each of the species, we found that synteny is very well conserved throughout the 12 *Drosophila* species for all three genes. ^a^The sequence for the *narya* homolog (not annotated) in *D*. *sechellia* was identified in a new release of the genome [68]. ^b^We identified evidence of a gene duplication of *vilya* in *D*. *ananassae*. The duplicated gene (*GF21626*) is intronless, suggesting it is a pseudogene. ^c^In *D*. *ananassae*, two genes were identified that could be potential distant homologs of *narya* and *nenya*, however neither one maintained synteny, and we therefore cannot determine the likelihood of which of the two genes is *narya* or *nenya*. Prior to the spilt of *D*. *ananassae* from the *melanogaster* subgroup, we failed to detect the *narya* homolog, suggesting the duplication of *nenya* likely occurred less than 40 Mya. Source of phylogeny tree: https://figshare.com/articles/Drosophila_25_species_phylogeny/5450602. (B) The evolutionary relationship of *narya* and *nenya* homologs. Both the potential *narya* and *nenya* homologs in *D*. *ananassae* cluster outside of *narya* and *nenya* nodes, and thus while they appear similar might not be true homologs. Scale bar indicates the number of nucleotide changes per site. (C) Protein alignment of Narya and Nenya using Jalview (http://www.jalview.org). Proteins were aligned and visualized with MUSCLE and ClustalX programs. Asterisks indicate the conserved residues in the C3HC4 RING finger domain. Residues corresponding to the predicted coiled-coil region are shown with a black line. Coiled-coil region was predicted by https://embnet.vital-it.ch/software/COILS_form.html [79]. The location of the frameshift in *narya*^*JJ6*^ is shown with an arrow, and the residues deleted in the *narya*^*G4*^ mutation are shown inside the black box.

We then investigated the conservation of *vilya*, *narya*, and *nenya* in the 12 fully sequenced genomes from the *Drosophila* Genomes Consortium. Using a tBLASTn search, we identified the most likely homolog in each of the 12 *Drosophila* genomes and determined if the gene locations maintained synteny. While we found evidence of *vilya* and *nenya* homologs across the *Drosophila* genus, we could not identify homologs of *narya* outside the *melanogaster* subgroup (Fig 1A). Maximum-likelihood phylogenetic analyses suggest that *narya* arose as a gene duplication event of *nenya* less than 40 Mya, prior to the separation of the *melanogaster* subgroup (Fig 1B). Within *D*. *melanogaster*, *narya* and *nenya* nucleotide sequences are 69.1% identical to each other, while Narya and Nenya protein sequences share only 49.1% identity and 66% similarity (Fig 1C). However, despite their high level of divergence, *narya* and *nenya* are evolving at a similar rate (S1 Table).

### *narya* and *nenya* are functionally redundant genes required for proper chromosome segregation during female meiosis

Given that *narya* and *nenya* are homologous to many of the Zip3 family members, we assessed whether these two genes had roles during meiosis. We had previously created several mutations in *narya* using TALEN-based mutagenesis where we specifically targeted the RING finger domain [42]. Since RING finger domains are known to mediate protein-protein interactions and are required for mediating E3 ligase activity, we speculated that mutations in this domain would abolish *narya* function. One such mutation resulted from an indel (insertion of 3 nucleotides/deletion of 13 nucleotides during repair) causing a frameshift at amino acid 42 that eventually truncates the protein to 115 amino acids. This truncated allele, known as *narya*^*JJ6*^, also lacks the last two conserved cysteines in the RING finger domain and therefore is likely nonfunctional (see Fig 1C).

Using FLP/FRT-mediated recombination with two *piggyBac* transposons that each flanked the *nenya* gene [43], we created a chromosomal deletion of *nenya* (*nenya*^*del*^). Because *nenya* is located within the intron of *minotaur*, a gene known to be required for silencing the piRNA pathway in oocytes [44], we also created an RNAi construct specific for *nenya* to assay its function in the absence of potential effects created by disrupting the *minotaur* gene. We used the GAL4-UAS system under the control of the *nanos* (*nos*) promoter (*Pnos-GAL4*::*VP16*) to induce expression of the *nenya* RNAi hairpin transgene (hereafter referred to as *nenya*^*RNAi*^). The *nos*GAL4::VP16-UAS system results in high levels of expression in the germline throughout most stages of oogenesis, including the germarium where meiosis begins [45,46,47]. qPCR analysis indicated that the *nenya* transcript levels were reduced by at least 50% in whole ovaries when *pValium22-nenya*^*RNAi*^ was driven within the germline (S2 Fig).

We tested each of these mutant alleles, individually and in combination with each other, for effects on meiotic chromosome segregation (Table 1). There was at most a weak effect on meiotic nondisjunction compared to controls for the homozygous mutants when tested individually. *narya*^*JJ6*^ showed low (2.2%), but statistically significant, levels of *X* chromosome nondisjunction when compared to the control (0.0%), while the *nenya* mutant (*nenya*^*RNAi*^) repeatedly showed wild-type chromosome segregation (0.3% *X* chromosome nondisjunction). In addition, there was no significant effect on meiotic segregation when there was only one copy of wild-type *narya* in the complete absence of *nenya* (0.5% *X* chromosome nondisjunction), suggesting that *narya* is not haploinsufficient as has been reported for members of this group in other species [22,48,49]. In contrast, double mutants (either *narya*^*JJ6*^; *nenya*^*del*^ or *narya*^*JJ6*^; *nenya*^*RNAi*^) showed high levels of *X* chromosome nondisjunction (49.0% and 32.4%, respectively), indicating that these genes have redundant functions. Supporting this proposal, we were able to rescue the nondisjunction phenotype in the *narya*^*JJ6*^; *nenya*^*del*^ double mutant with expression of a *narya*:*gfp* transgene in the germline (0.0% *X* chromosome nondisjunction) (Table 1).

**Table 1:** Narya and Nenya have redundant meiotic functions required for normal chromosome segregation.

Genotype	Copies of *narya* gene	Expression of *narya* rescue construct	Copies of *nenya* gene	Knockdown of *nenya* by RNAi	% *X* ND^a^	Adj total progeny scored	Statistical difference compared to control
*nosGAL4*/*+* (control)	+/+	—	+/+	no	0.0	2468	—
*nosGAL4 narya*^*JJ6*^/*narya*^*JJ6*^	-/-	—	+/+	no	2.2	2060	***
*nosGAL4*/*+*; *nenya*^*RNAi*^/*+*	+/+	—	+/+	yes	0.3	3790	n.s.
*nosGAL4 narya*^*JJ6*^/*narya*^*JJ6*^; *nenya*^*RNAi*^/*+*	-/-	—	+/+	yes	32.4	1813	***
*narya*^*JJ6*^/*nosGAL4*; *nenya*^*del*^/*nenya*^*del*^	+/-	—	-/-	—	0.5	391	n.s.
*narya*^*JJ6*^/*narya*^*JJ6*^; *PUASp-narya*^*GFP*^/*+*; *nenya*^*del*^/*nenya*^*del*^	-/-	no	-/-	—	49.0	557	***
*nosGAL4 narya*^*JJ6*^/*narya*^*JJ6*^; *PUASp-narya*^*GFP*^/*+*; *nenya*^*del*^/*nenya*^*del*^	-/-	yes	-/-	—	0.0	1269	n.s.

^a^ ND, nondisjunction

Females of the above genotypes were crossed to *y sc cv v f·car* / *B*^*s*^*Y* males. This cross allows for the identification of normal offspring (*XX* females, *XY* males), diplo-*X* and nullo-*X* exceptions. The table shows the summed nondisjunction frequency (% *X* ND). The total number of progeny scored are adjusted to account for the inviable progeny class (Adj total, see Methods).

***P<0.001, n.s. is *P*>0.001 and not significantly different to control with the number of progeny scored. Statistical test described in [50].

### Narya and Nenya are required for DSB formation

**Fig 2:**
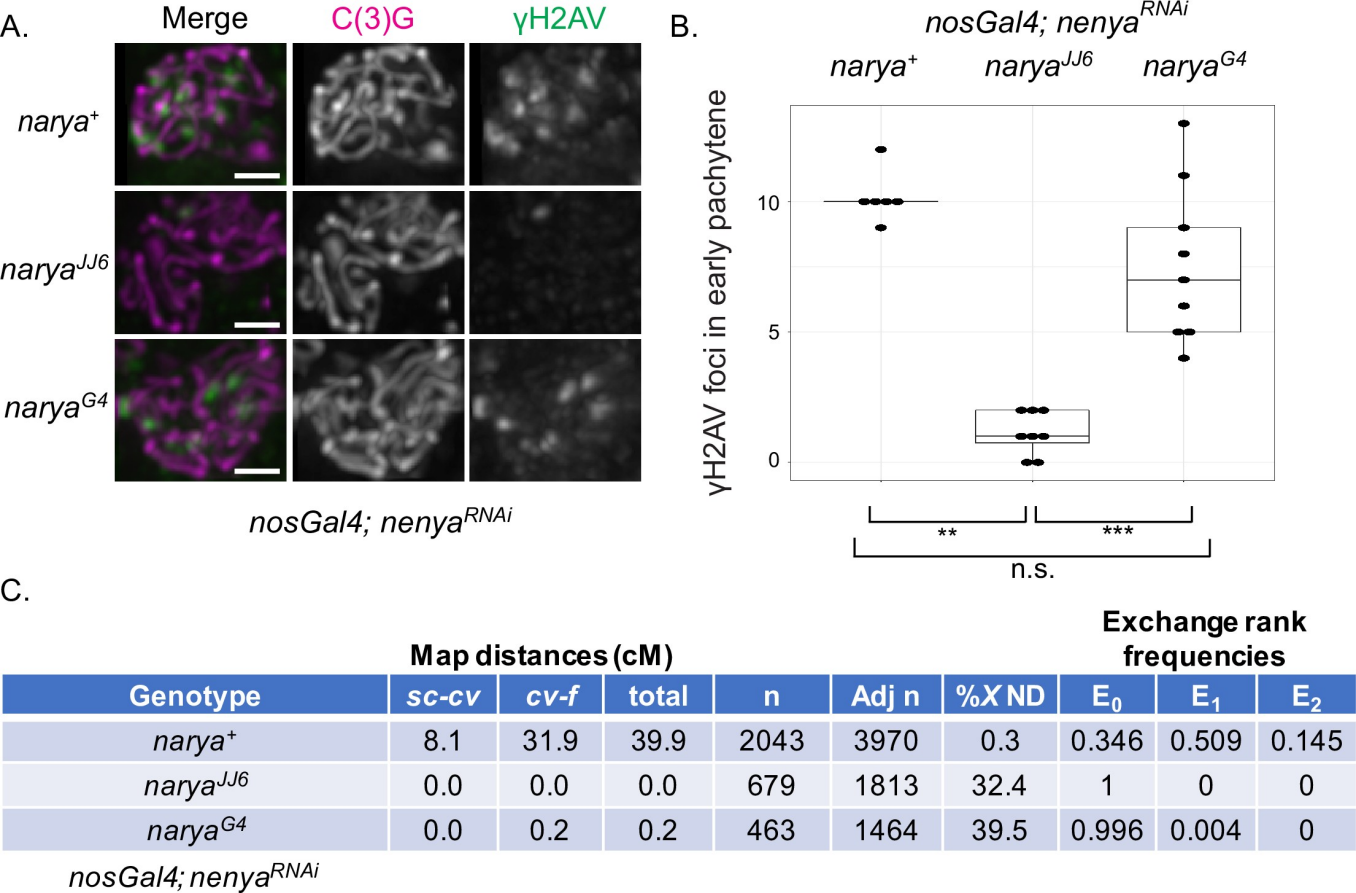
*narya* and *nenya* are required for DSB formation and function in the process of crossing over. (A) Early pachytene (region 2A) oocytes stained with C(3)G (magenta) to mark the SC and γH2AV (green) to mark the DSBs in *narya*^*+*^ (wildtype), *narya*^*JJ6*^ (null) or *narya*^*G4*^ (RING mutant) in the absence of *nenya* using the RNAi transgene expressed with the *nosGAL4* driver. Images are maximum-intensity projections of the deconvolved *z*-series through the selected nuclei. Scale, 1 μm. (B) Quantification of the number of γH2AV foci per region 2A early pachytene pro-oocyte in the genotypes from (A). Pro-oocytes in late region 2A were scored. DSB numbers for *nenya*^*RNAi*^ females in the presence of wild-type *narya* (mean 10.2 DSBs, SD ± 0.90, median 10) are consistent with wild-type oocytes in this region (10.8 DSBs) [54]. In the *narya* ^*JJ6*^*; nenya*^*RNAi*^ double mutant, the DSB numbers were severely reduced (mean 1.1 DSBs, SD ± 0.78, median 1). In the *narya*^*G4*^*; nenya*^*RNAi*^ double mutant, DSBs were formed but were slightly reduced compared to *nenya*^*RNAi*^ alone (mean 7.6 DSBs, SD ± 2.83, median 7). Number of pro-oocytes scored are *narya*^*+*^ (n = 6), *narya*^*JJ6*^ (n = 8) or *narya*^*G4*^ (n = 9). The box and whisker plot was created in RStudio using ggplot2. Each point represents the number of γH2AV foci scored within an SC-positive nucleus. The box indicates the upper and lower quartiles and the horizontal line indicates the median. The number of γH2AV foci in *narya* ^*JJ6*^*; nenya*^*RNAi*^ is significantly different than both *narya*^*+*^*; nenya*^*RNAi*^ and *narya*^*G4*^*; nenya*^*RNAi*^, while *narya*^*+*^*; nenya*^*RNAi*^ and *narya*^*G4*^*; nenya*^*RNAi*^ are not significantly different from each other (*P* = 0.077) (statistical test, two-tailed Mann-Whitney test). ***P<0.001, **P<0.01; n.s., statistically not significant. (C) *narya*; *nenya* double mutants are defective in meiotic recombination compared to *nenya*^*RNAi*^ alone, as assayed in the female progeny of the maternal genotype for intervals *sc*-*cv* and *cv*-*f* on the *X* chromosome. No recombinant *X* chromosomes were recovered for the *narya*^*JJ6*^; *nenya*^*RNAi*^ double mutant in either interval, and only one recombinant *X* chromosome (between *cv-f*) was recovered for the *narya*^*G4*^; *nenya*^*RNAi*^ double mutant. The exchange rank frequencies are shown (*E*_*0*_, no crossovers; *E*_*1*_, single crossovers; *E*_*2*_, double crossovers). The absence of recombination in the double mutants resulted in elevated levels of meiotic nondisjunction. Analysis by methods described in [50] reveals there is no significant difference in the nondisjunction rate (% *X* ND) when comparing the double mutants to each other with the number of progeny scored (*P*>0.001). Even though the *narya*^*G4*^; *nenya*^*RNAi*^ double mutant was able to induce DSBs (B), the vast majority of those DSBs were converted into noncrossovers. This figure shows the summed map distances in centiMorgans, and the summed nondisjunction levels. (n) is the number of female progeny scored from the maternal genotype listed in the recombination analysis. Adjusted n (Adj n) was the total progeny scored for both the recombination and nondisjunction assays done simultaneously. Adj n accounts for the inviable progeny class (see Methods).

In *vilya* mutants, the increase in meiotic nondisjunction is a result of failed initiation of the meiotic recombination process. To determine if the meiotic nondisjunction we observe in *narya nenya* double mutants occurs through a similar mechanism, we assayed the presence of DSBs formed in the pro-oocytes during pachytene (Fig 2). To do this, we used a phospho-specific antibody against the histone 2A variant (γH2AV). Phosphorylation of H2AV is an evolutionarily conserved rapid response that occurs at DSB sites [51,52,53]. We found that in the absence of only *nenya*, DSBs are formed at wild-type levels in early pachytene pro-oocytes (mean 10.2 DSBs, SD ± 0.90 compared to 10.8 DSBs in the same meiotic stage in a wild-type background [54]), consistent with the normal levels of chromosome disjunction (Fig 2A and 2B and S3 Fig). However, in the *narya*^*JJ6*^*; nenya*^*RNAi*^ double mutant, meiotic recombination failed to initiate in early pachytene cysts, with few, if any, DSBs detected (mean 1.1 DSBs, SD ± 0.78) (Fig 2A and 2B and S3 Fig). Similar results were obtained when analyzing γH2AV foci number in the *narya*^*JJ6*^*; nenya*^*del*^ double mutant (average 0.74 DSBs, SD ± 0.97 in 27 pro-oocytes), indicating that the level of RNAi knockdown for *nenya* transcript (less than 50% of wild-type *nenya* transcript levels within the whole ovary) was sufficient to mimic the genomic *nenya* deletion with regard to DSB formation function (see Materials and Methods).

We also failed to detect crossovers when assaying crossover formation using genetic markers along the *X* chromosome (Fig 2C). The failure to detect meiotically induced DSBs using the γH2AV antibody is not due to a general defect in modifying the histones at the DSB sites, as we can detect γH2AV foci during the endoreduplication cycle (S3 Fig). In addition, these effects on DSB formation are unlikely to be caused by defects in synaptonemal complex formation or in the selection of the oocyte by early-mid pachytene, as these processes appeared to be normal in the absence of *narya* and *nenya* (S3 Fig).

### *narya*^*G4*^ is a separation-of-function mutant showing that Narya is required for the process of crossing over

In the *narya* TALEN-based mutagenesis described above, we also created a second allele (*narya*^*G4*^) that deletes five amino acids, including the last cysteine in the RING finger domain, one amino acid prior to it, and the three amino acids that follow it (see Fig 1C). The reading frame in *narya*^*G4*^ is maintained after the deletion, thus this mutant likely expresses a form of the protein that is missing key residues to form the RING finger domain. We assayed whether *narya*^*G4*^, which lacks part of the RING finger domain, was able to facilitate DSB formation in the absence of *nenya*. We found that DSBs were formed in the *narya*^*G4*^; *nenya*^*RNAi*^ double mutant (mean 7.6 DSBs, SD ± 2.83), unlike in the *narya*^*JJ6*^; *nenya*^*RNAi*^ double mutant, indicating that DSB formation is not fully dependent on an intact RING finger domain of Narya (Fig 2B).

In the *narya*^*G4*^; *nenya*^*RNAi*^ double mutant, DSBs were induced at ~70% of the level observed for *nenya*^*RNAi*^ alone (Fig 2B), which led us to reason that we would see a decrease in the amount of nondisjunction (see Table 1) compared to the *narya*^*JJ6*^; *nenya*^*RNAi*^ double mutant that failed to form DSBs. Therefore, we assayed for both the level of nondisjunction and the presence of crossing over on the *X* chromosome in *narya*^*G4*^; *nenya*^*RNAi*^ double mutant females and compared that to the *nenya*^*RNAi*^ mutant and the double *narya*^*JJ6*^; *nenya*^*RNAi*^ mutant (Fig 2C). As expected, due to the severe reduction in DSBs in *narya*^*JJ6*^; *nenya*^*RNAi*^ females, we failed to recover any recombinant *X* chromosomes in their progeny (map distance of 0.0 cM, *E*_*0*_ frequency of 1.0). These females also showed high levels of *X* nondisjunction (32.4%) compared to *nenya*^*RNAi*^ alone, which makes wild-type levels of DSBs and disjoins homologous chromosomes properly (map distance 39.9 cM, *E*_*0*_ frequency of 0.346, 0.3% *X* nondisjunction).

We found that while the *narya*^*G4*^; *nenya*^*RNAi*^ mutant was able to form DSBs (see Fig 2B), those DSBs were not converted into crossovers (map distance of 0.2 cM, *E*_*0*_ frequency of 0.996), and females maintained high levels of *X* chromosome nondisjunction (39.5%) seen in the *narya*^*JJ6*^; *nenya*^*RNAi*^ mutant (Fig 2C). Although the frequency of *X* chromosome nondisjunction in the *narya*^*G4*^; *nenya*^*RNAi*^ females was greater than what was observed in the DSB-deficient *narya*^*JJ6*^; *nenya*^*RNAi*^ females, this difference is statistically not significant with the number of progeny scored, and both are consistent with published data for mutants that fail to form crossovers due to the absence of either DSBs or homologous chromosome synapsis (S2 Table) [55].

The failure to form crossovers was not due to a global defect in DSB repair, as we did not detect any delay in removal of the γH2AV mark at mid-pachytene (S4A Fig). We also failed to detect any defect in karyosome structure, such as a fragmented karyosome, that is typical of DNA repair mutants (S4B Fig) [56]. In addition, the fertility did not decrease from that of the *narya*^*JJ6*^; *nenya*^*RNAi*^ double mutant (each double mutant combination yielded ~19 progeny per female in the recombination assay). These data suggest that the *narya*^*G4*^ allele is a separation-of-function mutant that maintains the ability to form DSBs, albeit at reduced numbers, but causes a deficiency in the ability to repair those DSBs into crossovers. This also predicts a direct function of Narya in the formation of crossovers, in addition to its separable role in DSB formation.

### Narya localizes to sites of DSBs

Since the presence of either *narya* or *nenya* is required for DSB formation, and Narya is functionally redundant with Nenya, we next asked whether Narya localized to sites of DSBs. We analyzed the localization of Narya during pachytene by creating a green fluorescent protein (GFP)-tagged version of *narya* at the genomic locus using CRISPR/Cas9 technology. We tested the *narya*^*GFPcrispr*^ alone and in combination with both *nenya* alleles to determine if the *narya*^*GFPcrispr*^ allele was completely functional. Females that were homozygous for *narya*^*GFPcrispr*^ in the absence of *nenya* (either *nenya*^*del*^ or *nenya*^*RNAi*^) showed little to no meiotic chromosome segregation errors (S3 Table), indicating that *narya*^*GFPcrispr*^ is fully functional.

**Fig 3:**
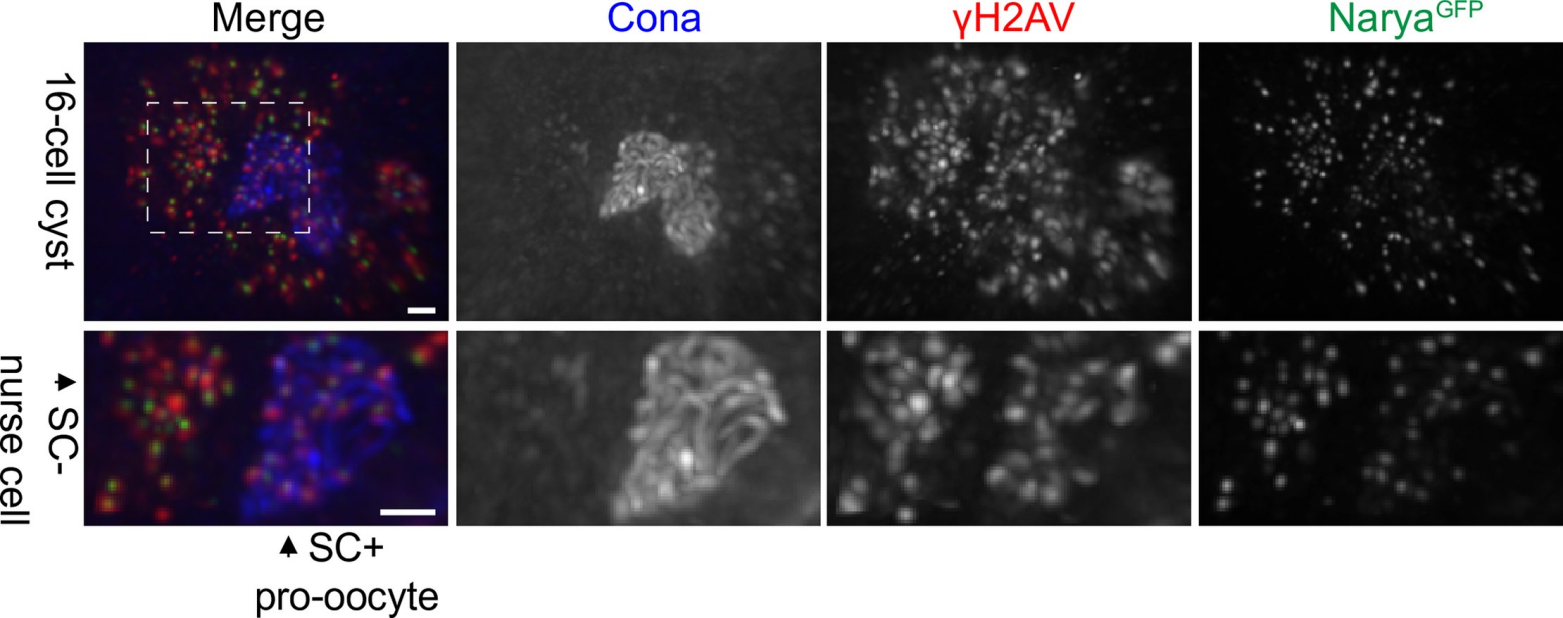
Narya localizes to sites of DSBs in both nurse cells and pro-oocytes. An early pachytene 16-cell cyst from the genotype *narya*^*GFPcrispr*^ stained with antibodies to Cona (blue) to mark the pro-oocytes, γH2AV (red) to mark the DSBs and GFP (green) to mark Narya. The dashed box indicates two nuclei that are enlarged in the bottom row. One is a nurse cell (SC-negative) and the other is one of the pro-oocytes within the cyst (SC-positive). Scoring the SC-positive pro-oocytes at early pachytene, we found that 93% of the Narya foci colocalized with γH2AV foci (10 pro-oocytes scored). Genotype of the image shown is *narya*^*GFPcrispr*^; *+*/*Sp*. Images are maximum-intensity projections of the deconvolved *z*-series through the selected nuclei. Scale, 1 μm.

**Fig 4:**
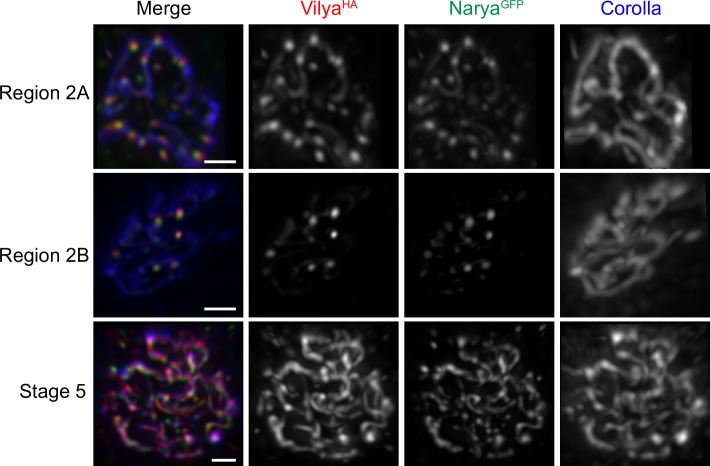
Narya and Vilya colocalize through pachytene, suggesting that Narya is a likely component of the RN. Immunofluorescence analysis of the colocalization of Vilya and Narya in *narya*^*GFPcrispr*^/ *nosGAL4 narya*^*GFPcrispr*^; *PUASp-vilya*^*3XHA*^*/+* oocytes throughout pachytene. Ovaries were stained with antibodies to HA (red) to mark Vilya, GFP (green) to mark Narya, and Corolla (blue) to mark the SC. Region 2A pro-oocytes are in early pachytene, Region 2B oocytes are in early-mid pachytene and Stage 5 oocytes are in mid pachytene. Images are maximum-intensity projections of the deconvolved *z*-series though the selected nuclei. Scale, 1 μm.

Immunofluorescence studies on whole ovaries showed that *narya*^*GFPcrispr*^ is highly expressed at the same stage in which DSBs are induced, as both a haze (detected in undeconvolved images) and faint staining along the SC with predominant numerous foci that decrease in number as the cysts progress through pachytene (S5 Fig and see below). Further analysis indicated that Narya^GFP^ foci colocalized with γH2AV foci, the histone modification created at the DSB site (Fig 3). These results are similar to our observation that Vilya also localizes to DSBs [16]. However, although Vilya, when overexpressed, colocalizes to ~60% of the γH2AV foci, Narya^GFP^ colocalized with γH2AV foci 93% of the time when expressed at the endogenous level (S6 Fig). In the 10 nuclei analyzed in early pachytene, the average number of DSBs was 13.1 and the average number of Narya^GFP^ foci was 10.6. In addition, since Narya^GFP^ is expressed from its endogenous promotor, we could determine that Narya^GFP^ also localized to the DSBs that are induced in the nurse cells within the 16-cell interconnected cyst. The number of Narya^GFP^ foci in the oocyte nuclei decreased as the cyst moved from early pachytene stage into early-mid pachytene (Region 2B) (see Fig 4), where the average number of Narya^GFP^ foci was 5.4 in the 12 nuclei analyzed. The number of Narya foci is similar to the number of Vilya^HA^ foci (4.8 foci) previously found at this stage [16], both of which are consistent with the number of crossovers formed per female meiosis.

Since Narya^GFP^ associated with DSB sites and the number of Narya^GFP^ foci decreased as pachytene progressed, we reasoned that these Narya^GFP^ foci might colocalize with Vilya foci. Therefore, we analyzed the localization of Narya^GFP^ in ovaries expressing *vilya*^*HA*^ in the germline using the *nos-GAL4*/*UAS* system (Fig 4). We found that Vilya^HA^ and Narya^GFP^ colocalized in SC-positive cells and remained colocalized as both types of foci decreased in number from early pachytene to early-mid pachytene (Region 2A to Region 2B). Examination of single-gallery *z*-slices throughout an early pachytene nucleus shows the faint localization of Narya^GFP^ to the SC and the association of Narya^GFP^ foci with Vilya^HA^ foci (S7 Fig). The maintenance of colocalization in early-mid pachytene (Region 2B, see Fig 4), a stage where Vilya^HA^ localizes to RNs by immuno-EM [16], demonstrates that Narya is a component of the RN.

Previous studies using high-resolution imaging followed by straightening of each of the chromosome arms have shown that at early-mid pachytene, the localization of Vilya^HA^ foci are consistent with both the number and position of crossovers, with each stretch of euchromatic SC between homologous chromosome arms primarily containing one Vilya^HA^ focus [16]. Taken together these results suggest that Vilya and Narya localize to the majority of the DSBs in early pachytene, and as the cyst progresses to early-mid pachytene, both proteins are maintained and concentrated at DSB sites destined to become crossovers. In addition, as we saw in earlier studies with Vilya, at late pachytene (Stage 5) when γH2AV foci are no longer present, there is a change in the localization of Narya^GFP^ from the discrete foci found at early pachytene to threads of staining exclusively along the SC, where it colocalizes with Vilya^HA^ (Fig 4, see Discussion).

### Localization of Narya to discrete foci is dependent on DSB formation, and the number of Narya foci does not change in the absence of DSB repair

**Fig 5:**
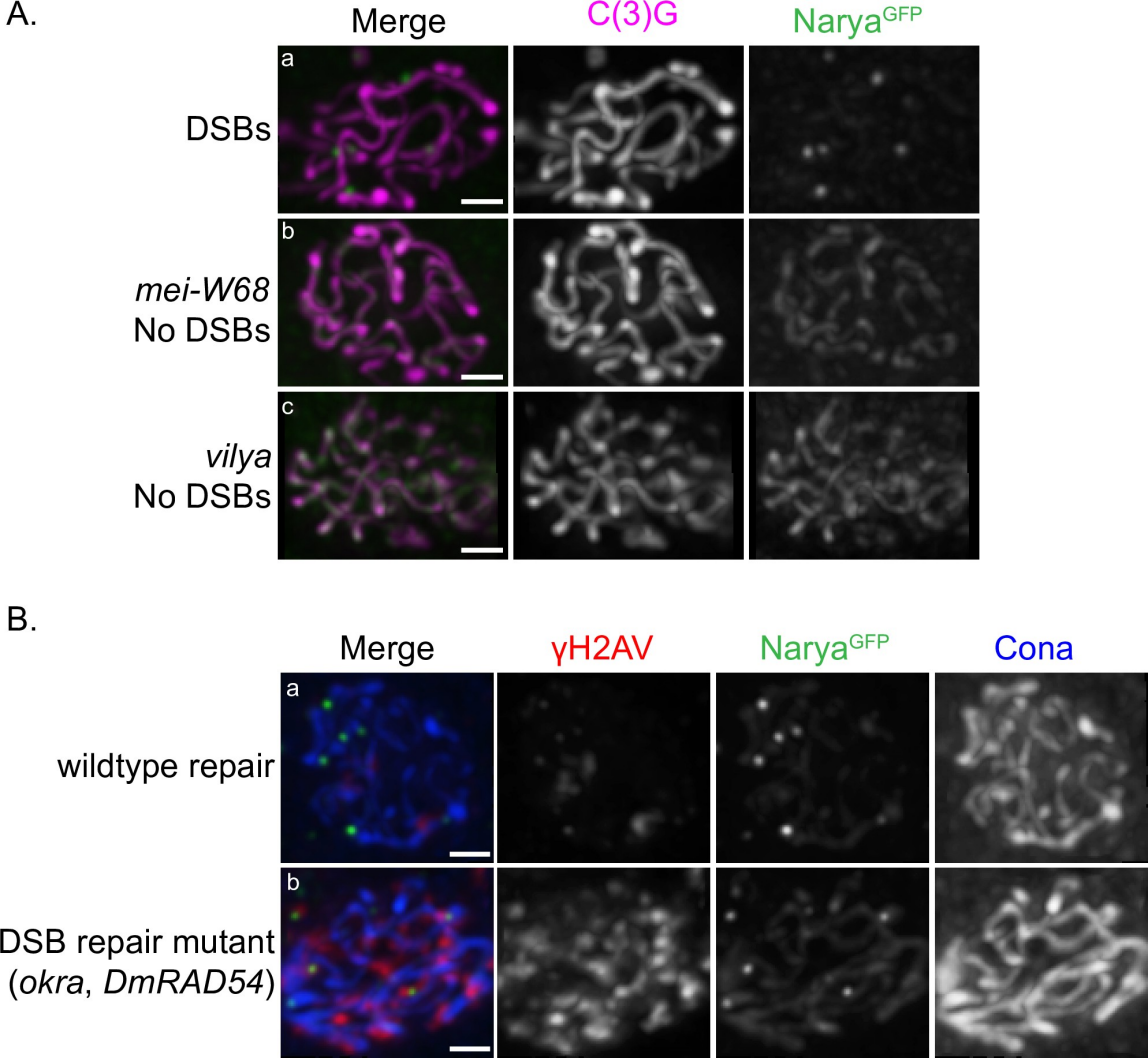
Narya’s localization to discrete foci is dependent on DSB formation, and the number of Narya^GFP^ foci do not increase in early-mid pachytene in the absence of DSB repair. (A) Early-mid pachytene (Region 2B) oocytes stained with C(3)G (magneta) to mark the SC and GFP (green) to mark Narya’s localization in the presence (a) or absence of DSBs (b and c). Genotypes are (a) *narya*^*GFPcrispr*^; *mei-W68*/*+*, (b) *narya*^*GFPcrispr*^; *mei-W68* and (c) *vilya narya*^*GFPcrispr*^. Images are maximum-intensity projections of the deconvolved *z*-series through the selected nuclei. Scale, 1 μm. (B) Early-mid pachytene (Region 2B) oocytes stained with Cona (blue) to mark the SC, γH2AV (red) to mark the DSBs, and GFP (green) to mark Narya in (a) *narya*^*GFPcrispr*^; *+*/*+* or (b) *narya*^*GFPcrispr*^; *okra*. The average number of Narya foci in (a) was 5.4 (n = 12) and in (b) was 5.0 (n = 6), with (n) being the number of pro-oocytes scored in Region 2B. Images are maximum-intensity projections of the deconvolved *z*-series through the selected nuclei. Scale, 1 μm.

Based on the number and localization of the Narya^GFP^ foci at sites of DSBs and the fact that the number of these foci decreased as DSBs were repaired into crossovers, we asked what effect DSB formation (Fig 5A) and/or lack of DSB repair (Fig 5B) had on the localization and number of Narya^GFP^ foci. As is also true for Vilya, Narya^GFP^ fails to form discrete foci and instead localizes along the SC when DSBs are absent (either in the absence of *mei-W68* or in the absence of *vilya*). However, in the absence of DSB repair, as in an *okra* (DmRAD54) mutant, Narya^GFP^ foci form, and the foci number in early-mid pachytene is similar to when DSB repair is normal. These results indicate that Narya displays two types of staining patterns depending on the presence or absence of DSBs. First, in the presence of DSBs, Narya forms discrete foci at DSB sites. Moreover, if there is a failure to repair those DSBs, there is not an increase in number of Narya foci at early-mid pachytene. We interpret this data to mean there is not an increase in the number of designated crossover sites in the absence of DSB repair. Second, in the absence of DSBs, either because the DSBs are undergoing normal repair or fail to form, Narya displays thread-like SC staining.

### Yeast two-hybrid analysis indicates that Narya, Nenya and Vilya can physically interact

Since Narya and Vilya colocalize at sites of DSBs and crossovers, and Narya and Nenya are functionally redundant, we wanted to determine if Nenya can physically associate with Narya and/or Vilya. Due to the lack of a functional *nenya* epitope-tagged transgene or antibodies to any of the RING finger domain proteins, we used the yeast two-hybrid system to help us understand the associations and/or interactions between these three proteins.

**Fig 6:**
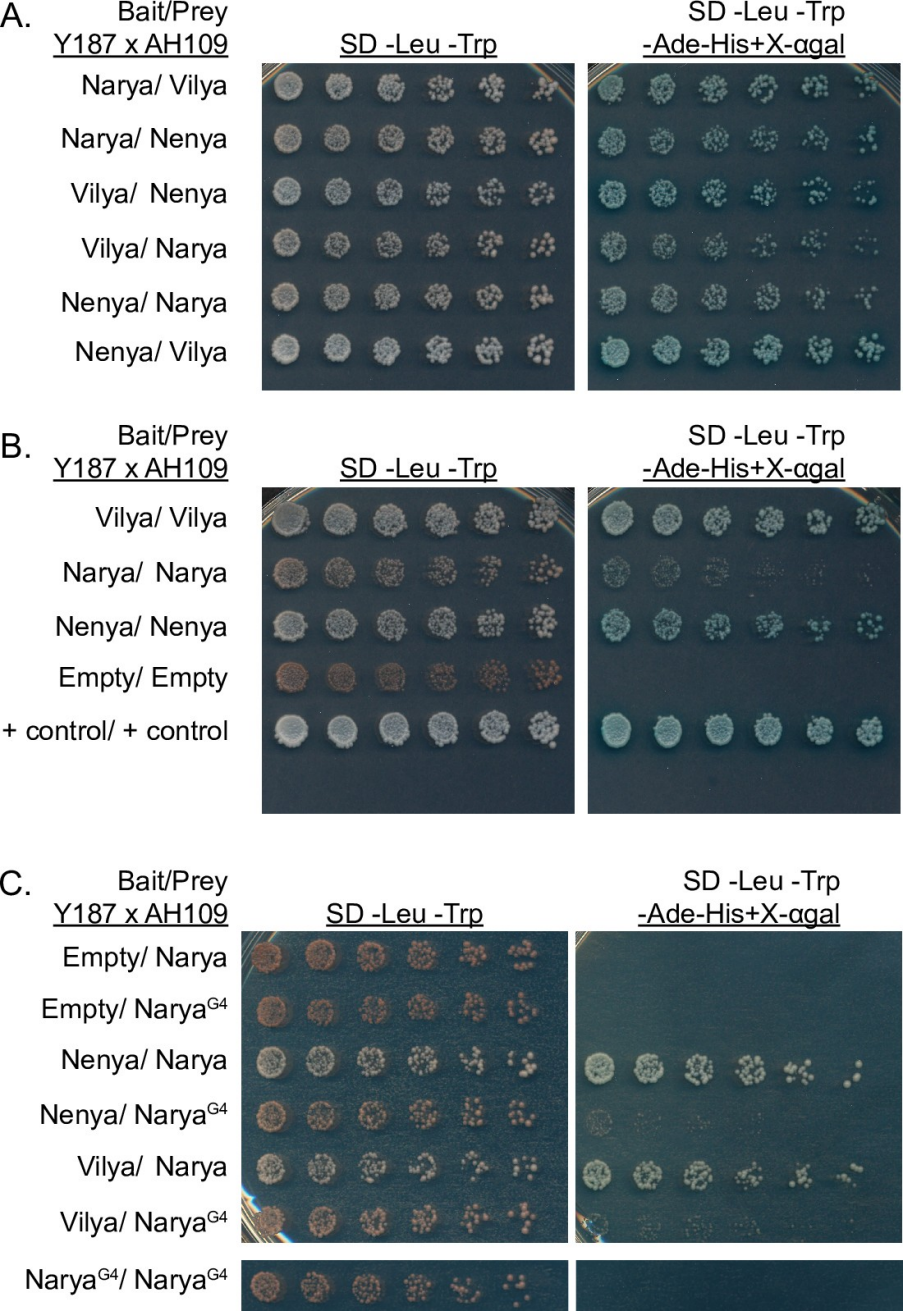
Yeast two-hybrid studies suggest that Vilya/Narya/Nenya likely interact with each other and themselves and may do so through the RING finger domain of Narya/Nenya. (A) Narya, Nenya and Vilya interact by yeast two-hybrid in all pairwise combinations in both directions. (B) All three RING finger proteins interact with themselves. Narya was able to interact with itself, although not as robustly as Nenya:Nenya or Vilya:Vilya. Control plasmids were supplied by Clontech (pGBKT7-53 and pGADT7-T). (C) Narya^G4^ was unable to interact with Nenya and Vilya, and it also failed to interact with itself. Confirmation of Narya^G4^ expression is in S10 Fig. In each experiment, six twofold dilutions of equal starting amounts were plated on each of the selection plates.

We cloned *narya*, *nenya* and *vilya* into yeast two-hybrid vectors and tested their ability to interact with each other in all pairwise combinations. In addition, we tested for the ability of each protein to interact with itself (Fig 6). We found that Narya, Nenya and Vilya interact with each other (Fig 6A) as well as with themselves (Fig 6B).

Previous studies showed that both the RING finger domain of Vilya as well as its C-terminal region are required for its interaction with the DSB accessory protein MEI-P22 [16], so we further investigated the interaction of Vilya with Narya and Nenya by testing whether C-terminal and RING finger domain mutants of Vilya could still bind to Narya and Nenya. Neither Vilya’s RING finger domain nor it’s C-terminal region were required for its interaction with either Narya or Nenya (S8 Fig), indicating that Vilya likely interacts with Narya and Nenya through the middle region of the Vilya protein, perhaps assisted by the coiled-coil domain. Additionally, although Vilya interacts with MEI-P22 as well as Narya and Nenya, neither Narya nor Nenya were able to interact with MEI-P22 by yeast two-hybrid (S9 Fig).

Finally, since Narya interacted with both Nenya and Vilya, we then tested the ability of Narya^G4^ to interact with each of these proteins (Fig 6C). We found that Nenya-Narya^G4^ and Vilya-Narya^G4^ binding were substantially reduced compared to binding with wild-type Narya protein. We also found that Narya^G4^ was unable to interact with itself. This inability of Narya^G4^ to strongly interact with Nenya, Vilya or itself is not due to the lack of expression of Narya^G4^ (S10 Fig). When considered with the results above showing that Narya and Vilya colocalize, these yeast two-hybrid data indicate that all three proteins likely function as part of the RN.

## Discussion

### RING finger domain-containing proteins localize to crossover sites

The finding of three Zip3 family members in *Drosophila*, Narya, Nenya and Vilya, is consistent with studies in other organisms that show that the presence of multiple Zip3 homologs within an organism is not uncommon [25,48,57]. These proteins share common structural features such as a RING finger domain near the N-terminus, and in those organisms that form SC, a predicted coiled-coil domain within the middle third of the protein (reviewed in [31] and [23]). The presence of a RING finger domain suggests that these proteins play roles in either the ubiquitination or sumoylation pathway as E3 ligases [58]. Indeed, members of this family have been shown to be required for sumoylation (e.g., Zip3 [33,37], RNF212 [59]) as well as ubiquitination (e.g., HEI10, mammals [36,59]) or are speculated to be a sumoylation/ubiquitination switch (e.g., HEI10, Sordaria [20]) necessary to stabilize and/or promote crossover formation. However, the mechanism(s) by which the *Drosophila* homologs act is currently unknown.

Studies in a number of organisms have shown that Zip3-like proteins function as pro-crossover factors during meiosis and localize along the SC as linear arrays of foci and/or as discrete foci at crossover sites (reviewed in [31]). We provide evidence that at least two of the RING finger domain-containing proteins in *Drosophila*, Narya (this study) and Vilya [16], also localize in this manner. Using an overexpression construct, we previously showed that Vilya localizes along the central region of the SC and at sites of DSBs. Eventually Vilya becomes concentrated at crossover sites, as immuno-EM studies demonstrated that Vilya localizes at RNs. In this study, we analyze the localization pattern of Narya using a CRISPR/Cas9-engineered epitope-tagged version of *narya* at the genomic locus, which eliminates many of the caveats of using an overexpression system. Although very faint Narya SC staining could be seen when analyzing *narya*^*GFPcrispr*^, the predominant staining was discrete foci that localized to the majority of DSBs early in pachytene, and those foci decreased in number as pachytene (and DSB repair) progressed (see Fig 4). Vilya has also been shown to localize to a subset of DSBs during early pachytene [16]. The discrete Narya foci observed in both early and early-mid pachytene colocalized with Vilya, indicating that these two proteins are found together within the SC at DSBs as they form and are repaired. These findings indicate that Narya is also found at crossover sites and is a component of the RN. The identification of two *Drosophila* Zip3-like proteins at sites of maturing crossovers is consistent with studies of all other homologs in that they also localize at or associate with proteins known to be at crossover sites [19,20,22,25,32,37,38,48,60].

### RING finger domain-containing proteins in *Drosophila* play a role in DSB formation

The similarities in localization of both Narya and Vilya to other Zip3 family members predict these proteins may play a role in crossover control. However, our previous studies and those described here indicate that, in a fashion that is so far unique to *Drosophila*, Narya, Nenya and Vilya first function prior to DSB fate determination; which is to say that they are essential for meiotic DSB formation. Our data demonstrate that *narya* and *nenya* encode functionally redundant proteins that are necessary for the induction of meiotic DSBs during early pachytene. Only in the absence of both gene products is there an increase in meiotic nondisjunction resulting from the lack of recombination due to the failed induction of DSBs. This severe reduction of DSB induction is not seen in mutants that affect the formation of the SC. Mutants that fail to form SC (*c(3)G*) or that form fragmented SC (*c(2)M*) do not eliminate DSBs but reduce their numbers in the pro-oocytes [61]. Therefore, we propose that Narya and Nenya play a direct role in the formation of DSBs. In addition, the absence of *vilya*, or other genes required for DSB formation (e.g., *mei-W68* or *mei-P22*), results in identical meiotic phenotypes [9,14,16,61]. However, because we are basing the lack of DSBs on the absence of γH2AV signal, we cannot rule out the possibility that *narya* and *nenya*, and possibly *vilya*, instead allow the very rapid repair of DNA lesions thereby reducing the number and/or amount of γH2AV signals, as has recently been found for RNF212 in female mouse oocytes [62].

Previous studies demonstrated that Vilya interacts with MEI-P22, the potential partner of DmSpo11, which is known to be required for DSB formation. In addition to the colocalization of Narya and Vilya throughout pachytene, yeast two-hybrid studies show that Narya, Nenya and Vilya all interact with each other. The direct interaction of Narya or Nenya with Vilya does not appear to require a functional N-terminal RING finger domain of Vilya, which was necessary for its interaction with MEI-P22, or its C-terminal region that is known to be required for DSB formation. This may indicate that it is the middle third of Vilya that is necessary for its interaction with Narya and Nenya. As the middle region of Vilya contains the predicted coiled-coil domain, a domain that can mediate protein-protein interactions, it is highly possible that these proteins interact through their coiled-coil domains. However, the observation that the RING finger domain mutant, Narya^G4^, failed to interact with itself, Nenya and Vilya in the yeast two-hybrid assay, may indicate that the coiled-coil domains are not sufficient for interaction and that the RING finger domain may also be required for protein-protein interactions. We should note that the mutations in the RING finger domain of Vilya used in this analysis differed from the mutation in Narya. The Vilya mutations were single amino acid substitutions, whereas the mutation in Narya resulted in a five amino acid deletion. It is possible that the deletion in Narya alters the protein structure, thus disrupting the ability of the coiled-coil domain to interact with other proteins.

Many proteins that localize to the SC contain coiled-coil domains, and our studies here show that while Narya primarily localizes to discrete foci, SC localization is observed at low levels in a *narya*^*GFPcrispr*^ background in early pachytene and Narya is exclusively found along the SC in late pachytene (Fig 4). The SC localization at early pachytene could be due to the propensity of coiled-coil proteins to localize to the SC, or this localization may be required for wild-type levels of DSBs, as most meiotic mutants that fail to assemble SC only induce DSBs at a reduced level [61]. In addition, we show that in the absence of DSB formation, discrete Narya foci fail to form and instead Narya localizes along the SC in a similar staining pattern to that of the SC protein C(3)G. The Narya SC localization occurs in the absence of either *mei-W68* or *vilya*, indicating that although Narya and Vilya colocalize and may interact directly, Narya’s localization to the SC is not dependent on Vilya. The exclusive localization of Narya to the SC during late pachytene in the presence of wild-type DSB repair was similar to the distribution of Vilya in the same genetic backgrounds. In this study, however, we were able to assess the localization of Narya at endogenous levels throughout pachytene, and therefore we are confident that there is a change in the localization pattern from foci in early-mid pachytene to linear staining along the SC in late pachytene. Currently, we do not understand the function of this redistribution. It is possible that Narya, and perhaps Vilya, play a role in the disassembly of SC that occurs post DSB repair.

### A separation-of-function mutation in *narya* links DSB formation to crossover formation

Based on the relationship of Narya to other Zip3 homologs and its localization and association with Vilya, which is found at RNs, it seems likely that Narya might have a role in processing DSBs into crossovers. However, the fact that *narya* and *nenya* are also required for DSB formation makes it difficult to analyze the role of either in crossover formation. An analogous problem arose when studying mutations that affected the function of *vilya* [16]. In that case, we reasoned that if Vilya could be recruited to exogenous DSBs from its localization along the SC when DSBs were absent, it would provide strong evidence that Vilya had a role in crossover formation. Using X-rays to produce exogenous DSBs, that is precisely what we found. In the absence of *mei-W68* (Dm Spo11), but following X-irradiation, Vilya, which in this background is found exclusively along the SC, formed discrete foci at a subset of exogenous DSBs. Here we provide direct evidence that Narya plays an essential role in the formation of crossovers. We obtained an in-frame deletion within the RING finger domain of *narya* (*narya*^*G4*^) and analyzed its role in DSB formation and crossing over in the absence of *nenya*. Unlike the null allele of *narya* (*narya*^*JJ6*^), *narya*^*G4*^ retained its ability to function in DSB formation (Figs 2 and S3). There was a slight decrease in the mean DSB number, and a wider range of DSBs in the nuclei assayed, but a significant number of DSBs (average of 70%) were formed. Surprisingly though, none of the DSBs that were formed were able to be converted into crossovers. The DSBs in *narya*^*G4*^; *nenya*^*RNAi*^ oocytes were most likely repaired as noncrossovers, given that we did not see either a karyosome fragmentation defect associated with the lack of DSB repair or a more severe fertility defect from the *narya*^*JJ6*^; *nenya*^*RNAi*^ females. The presence of DSB repair combined with the lack of crossovers resulted in high levels of nondisjunctional progeny. In summary, our data demonstrate that in *Drosophila*, members of the Zip3 family are required to both form DSBs and repair those DSBs into crossovers, and flies use a mechanism to ensure these processes are directly linked. Future studies will need to be done to determine the precise function of Narya and whether it acts to stabilize crossover intermediates and/or in the maturation of crossovers.

### The evolutionary relationship between *narya* and *nenya*

Based on sequence comparison, *narya* appears to have duplicated from *nenya* less than 40 million years ago, after the split of *D*. *ananassae* from the *melanogaster* subgroup. Both genes have been maintained in all the sequenced species of the *melanogaster* subgroup. We provide evidence that *narya* and *nenya* encode proteins that are functionally redundant with regard to their role in the early steps of meiosis. The preservation of both genes and their functional redundancy is surprising since genetic redundancy in *Drosophila* is not prevalent [63,64]. In fact, studies have shown that the vast majority of meiotic genes are not duplicated [65]. In addition to the duplication of *nenya* found in the *melanogaster* subgroup, we also found evidence of a gene duplication of *vilya* in *D*. *ananassae*. However, unlike the *vilya* homolog in *D*. *ananassae* that maintains synteny, the duplicated gene is intronless, likely caused by a retrotransposition event. Retrotransposed duplicates do not bring upstream and downstream regulatory regions with them and are often pseudogenized, as they are less likely to be expressed or maintained [66].

It is not obvious why the *melanogaster* subgroup has maintained two meiotic genes with the same function. As we presently lack any alleles that allow for visualization of Nenya, we can only speculate that Nenya is behaving exactly as Narya. However, we cannot rule out that the functional redundancy of these two genes is due to their roles in DSB formation, and that Narya may be more important than Nenya at the RN in the formation of crossovers. While we failed to detect any meiotic chromosome nondisjunction in the absence of *nenya*, we consistently observed low levels of chromosome segregation errors in the absence of *narya* (*X* chromosome nondisjunction levels ranging from 2–4%, see Table 1). We know based on their sequence alignment that the C-terminal region shows the least conservation. Perhaps future studies will determine whether this domain could be important for independent functions of the two proteins. Our studies here have shown that Narya’s RING finger domain is critical for crossing over but not for its role in DSB formation; it will be interesting to dissect these same domains in Nenya.

Taken together, these studies identify two functionally redundant genes, *narya* and *nenya*, that are required for the induction of meiotic DSBs. Both of these genes encode proteins that are structurally and functionally similar to the *Drosophila* protein Vilya, and all three show similarities to a family of proteins found in many organisms that are required to process meiotic crossover events. We show here that in addition to its role in DSB formation, Narya is required for crossover formation. While *Drosophila* may lack a subset of both DSB accessory and pro-crossover homologs present in the majority of model systems, flies have clearly found a way to utilize the proteins they do have for both processes.

## Materials and methods

### *Drosophila* genetics

*Drosophila* strains were maintained on standard food at 24°C. Descriptions of genetic markers and chromosomes can be found at http://www.flybase.org/. Stocks used in this study include *Pnos-GAL4*::*VP16* [45], *PUASp-vilya*^*3XHA*^ [16], *vilya*^*826*^ [16], *mei-W68*^*4572*^ [67], *narya*^*JJ6*^ [42], *Pnos-GAL4*::*VP16 narya*^*JJ6*^ (this study), *narya*^*G4*^ [42], *Pnos-GAL4*::*VP16 narya*^*G4*^ (this study), *nenya*^*del*^ (this study), *okra*^*AA*^ *cn bw*/*CyO* and *okra*^*RU*^ *cn bw*/*CyO* [56]. *vilya* refers to the genotype *vilya*^*826*^, *mei-W68* refers to the genotype *mei-W68*^*4572*^, and *okra* refers to the genotype *okra*^*RU*^/*okra*^*AA*^.

The rescue construct (below) and all alleles of *narya* generated in this manuscript were made using the Canton-S stock or the Canton-S *narya* sequence. The Canton-S *narya* sequence differs from the *narya* sequence on FlyBase at 10 bases. Nine of these base changes encode for the same amino acid. One of the base changes result in an amino acid change from alanine at position 69 in FlyBase to glutamic acid in the Canton-S stock. *narya*^*JJ6*^ and *narya*^*G4*^ were generated using TALEN mutagenesis as described in [42]. *narya*^*JJ6*^ deletes 16 bases, adds 3 and makes a nonsense allele after the lysine, and *narya*^*G4*^ removes 15 bases, causing the deletion of 5 amino acids (deletion CGQVL), but maintains the frame of the gene. *nenya*^*del*^ was generated by FLP/FRT recombination with two *piggyBacs* (*PBac{WH}CG5508[f01088]* and *PBac{WH}CG5508[f04927]*) that both reside in the intron of *CG5508*, which also contains *CG31053* (*nenya*).

### Identification of *narya*, *nenya* and *vilya* homologs

Coding sequences obtained from FlyBase for *D*. *melanogaster vilya*, *nenya* and *narya* were used as BLAST queries in order to retrieve homologous sequences for additional *Drosophila* species. The tBLASTn option was used with the expect threshold set to 0.05. Retrieved genes were then examined for shared synteny with *D*. *melanogaster*. For *narya* and *nenya* in particular, this was done by determining whether they were found within the introns of the homologs of *D*. *melanogaster minotaur* or *CG32533*, respectively. Originally, the *narya* homolog in *D*. *sechellia* could not be definitively determined due to the poor coverage in the area, although partial *narya* sequence could be found in the first intron of the *CG32533* homolog. With the recent release of a new *D*. *sechellia* genome [68], full sequence of a syntenic *narya* homolog was identified.

### Phylogenetic analyses

Nucleotide sequences for identified homologs were aligned using the PRANK_+F_ algorithm [69]. Maximum-likelihood trees were inferred using IQ-TREE [70], with the best-fit model selected by ModelFinder [71]. To infer the relative evolutionary rates of *narya* and *nenya*, Tajima’s relative rate tests [72] were performed using MEGA7 [73] on the PRANK-aligned nucleotide sequences.

### Constructing the *narya*^*GFP*^ genomic allele

A *narya*^*GFP*^ knock-in was generated using CRISPR/Cas9 technology. Using the CRISPR Optimal Target Finder (http://tools.flycrispr.molbio.wisc.edu/targetFinder/), two genomic regions were selected for making the gRNAs [CCTTCCACTTGACCCAGTGCCG**G** and AGATCTTCTCCGCGTTGACTG**G**G (the PAM sequences are underlined)] and were cloned into the pU6-BbsI-chiRNA vector (gift from Melissa Harrison, Kate O’Connor-Giles and Jill Wildonger; Addgene plasmid #45946) [74] by the protocol outlined at http://flycrispr.molbio.wisc.edu/protocols/gRNA using oligos (IDT) 5’-CTTCGCCTTCCACTTGACCCAGTGC-3’ and the complement 5’-AAACGCACTGGGTCAAGTGGAAGGC-3’ and 5’-CTTCGAGATCTTCTCCGCGTTGACT-3’ and its 5’-AAACAGTCAACGCGGAGAAGATCTC-3’, respectively. Plasmid DNA was isolated using a Qiagen Midi Prep Kit.

The homologous recombination repair template containing the *narya* gene with a 3’ GFP epitope tag with 1,000 bases of genomic sequence both up- and downstream of the *narya* gene was generated in the pBS-KS+ vector (Clontech) by the following method. Using the Canton-S stock as the genomic DNA source (gift from Dana Carroll), we first cloned in the region 5’ to the *narya* gene and the majority of the *narya* gene using primers 5’-[Phos]GTGGCGCATCGTTGTCAGTC-3’ and internal gene primer 5’-[Phos]CAGAAGGCATATCCGACGGC-3’ using the *EcoR*V site in pBS that was previously digested and dephosphorylated. The insertion of this fragment was sequenced for directionality so that the 3’ end of the *narya* gene was positioned closest to the *Xba*I site in the pBS vector. The pBS vector containing this piece of the genome was digested with *Stu*I (which cuts only within the *narya* gene) and *Xba*I (which cuts within the pBS vector). A *Stu*I/*Xba*I fragment that contained the end of the *narya* gene at the internal *Stu*I site through a 3’ in-frame GFP tag was amplified from pUASP-attB-*narya*^*GFP*^ (below) using primers 5’-GTATGCGGCCGGATGTTTCGAGTGCA-3’ and 5’-GCGCTCTAGATTACTTGTACAGCTC-3’ and then digesting with *Stu*I and *Xba*I and used to clone into the vector. The 1,000 bases of genomic region 3’ to the *narya* gene was then cloned into this vector using primers 5’-GCCGTCTAGATCACTCCAATTACTTG-3’ and 5’-GTACTCTAGACTGCGATCCTCGACAG-3’ and cloned into the *Xba*I site in the above vector. The insertion of this fragment was sequenced for directionality.

Following the creation of the homologous repair template, which consisted of 1,000 bases upstream of *narya*, the *narya* gene with cloned GFP tagged at the 3’ end of the gene and 1,000 bases downstream of *narya*, the two PAM sequences in the *narya* gene were mutated using the Quik Change II XL Site-Directed Mutagenesis Kit (Agilent Technology). The base changed in the PAM sequence is in bold above. In both cases, the codon remains unchanged.

250 ng of each gRNA plasmid and 500 ng of the homologous repair template plasmid were injected (BestGene) into *y w*; *nosCas9* (on II at attP40) (gift to BestGene from Shu Kondo). Potential CRISPR/Cas9 hits were screened with primers 5’-GTTGCAGCAGCTGGAGCAGA-3’ and 5’-GGTGAGTGCTCCCCAGATTG-3’, which amplify a region spanning the GFP insertion on the homologous repair template, allowing for PCR fragment size to visualize a repair off the homologous template. Once a CRISPR/Cas9 insertion was identified, the entire homologous region used in repair was sequenced. In this case, only one G0 fly had the correct insertion and was used for further analysis.

### Generation of rescue transgenes

pUASp-attB [75] *narya*^*GFP*^ was made by cloning the CDS of *CG12200* minus stop codon with primers 5’-GTATGCGGCCGCATGTTTCGAGTGCATTGCA-3’ and 5’-GTATGCGGCCGCCAAGACGAAAGCCTTTAGTG-3’ into a *Not*I digested pUASp-attB vector that previously had cloned in a *venus* (GFP) tag at *Not*I and *Xba*I. The CDS was sequenced for directionality.

### RNAi line and qPCR

An RNAi hairpin for *nenya* was identified using http://www.flyrnai.org/cgi-bin/RNAi_find_primers.pl. The sequence identified (GGACATAGATTGCCTTGAAGA) (underlined below) had no predicted off-targets and only shares five bases with *narya*. The hairpin was cloned using the oligos (IDT) 5’-CTAGCAGTGGACATAGATTGCCTTGAAGATAGTTATATTCAAGCATATCTTCAAGGCAATCTATGTCCGCG-3’ and 5’-AATTCGCGGACATAGATTGCCTTGAAGATATGCTTGAATATAACTATCTTCAAGGCAATCTATGTCCACTG-3’ into the *pValium22* vector (gift from Jian-Quan Ni and Norbert Perrimon), https://fgr.hms.harvard.edu/trip-plasmid-vector-sets.

qPCR determined that the level of *nenya* knockdown, when expressed in the female germline using the *nos-GAL4*::*VP16* driver, was greater than 50% of *nos-GAL4*/ +; *narya*^*RNAi*^/ + or Canton-S (wild-type) *nenya* transcript levels. While the *nenya* transcript levels are higher than what might be expected given the phenotype, this observation may be explained by the process in which the cDNA was synthesized. Since random hexamer primers were used to amplify cDNA from total RNA, we cannot rule out that the remaining levels of *nenya* transcript in the presence of RNAi knockdown are not from amplified, unspliced RNA from *minotaur* in which *nenya* resides. It is also possible that the remaining levels of *nenya* transcript are from expression of *nenya* in the somatic cells of the ovary, since the knockdown was specific to the germline. As well, based on published data of *nanos* RNA and protein, there are varying levels of expression in egg chambers of different stages within the ovariole [46].

The *narya* RNAi hairpin (GCAAGATCTCCAAGTTCCAAG), which had no predicted off-targets and differed from *nenya* sequence at three bases, was used as a non-specific RNAi control. Two qPCR *nenya* primer pairs were used to determine the relative level of transcript present in *nos-GAL4*/+; *nenya*^*RNAi*^/+ ovaries compared to Canton-S control ovaries. Total RNA from ovaries was isolated using the Promega Maxwell RSC Simply RNA Tissue Kit using standard protocol except for increasing the amount of DNase to 10 μL per sample. cDNA was synthesized from total RNA using the Invitrogen SuperScript III First-Strand Synthesis System for RT-PCR using random hexamers. Using the CAS qPCR Setup Robot to prepare the plates, each genotype was run in triplicate using Quanta Biosciences PerfeCTa SYBR Green FastMix ROX reagent. The *nenya* primer set was 5’-ACGTCGAGCCAACGTTGATC-3’ and 5’-TCGATCGGAATCGCTCGCAG-3’, and the control transcript primer set used was 5’-TGGACAGGTCATCACCATCGGAAA-3’ and 5’-TTGTAGGTGGTCTCGTGAATGCCA-3’ for ACT42A (FBgn0000043).

### Meiotic nondisjunction and recombination assays

The frequencies of meiotic nondisjunction and meiotic recombination on the *X* chromosome were measured by crossing single virgin females of the listed genotypes to *y sc cv v f·car / B*^*s*^*Y* males. This cross allows for the recognition of nondisjunctional offspring from the mother as *B*^*s*^ females (diplo-*X* exceptions) and *B*^*+*^ males (nullo-*X* exceptions). Normal segregation results in *B*^*+*^ females and *B*^*s*^ males. Nondisjunction frequency is calculated as the sum of exceptional progeny X 2 (to correct for the inviability of triplo-*X* and nullo-*X* exceptional progeny) divided by the sum of all progeny classes (viable plus inviable; denoted as adjusted total progeny scored). For *X* recombination analysis, only the female progeny (denoted as n) were analyzed for the intervals *sc*-*cv* and *cv*-*f*. *y* and *v* markers were unable to be scored due to the presence of *y+* and *v+* in the *PUASp-nenya*^*RNAi*^ transgene inserted at *attP40*.

### Yeast two-hybrid

Yeast transformation, mating and two-hybrid assays were done according to The Matchmaker Gold Yeast Two-Hybrid System User Manual (Clontech). AH109 yeast were used in place of Y2Hgold. The AH109 genotype is as follows: *MATa*, *trp1-901*, *leu2-3*, *112*, *ura3-52*, *his3-200*, *gal4Δ*, *gal80Δ*,*LYS2* : : *GAL1*_*UAS*_*-GAL1*_*TATA*_*-HIS3*, *GAL2*_*UAS*_*-GAL2*_*TATA*_*-ADE2*, *URA3* : : *MEL1*_*UAS*_*-MEL1*_*TATA*_*-lacZ*. Y187 genotype is as follows: *MATα*, *ura3-52*, *his3-200*, *ade2-101*, *trp1-901*, *leu2-3*, *112*, *gal4Δ*, *met–*, *gal80Δ*, *URA3* : : *GAL1*_*UAS*_*-GAL1*_*TATA*_*-lacZ*. cDNAs were cloned into either the pGADT7 or the pGBKT7 prey and bait vectors using restriction sites within the vector and contained within the PCR primers. The CDS for *narya* and *nenya* were obtained from Canton-S, as these genes do not contain introns.

Western blot analysis from yeast haploid cells was performed as described in [16].

### Immunohistochemistry

Germarium preparation for whole-mount immunofluorescence was performed as described in [16]. Primary antibodies used included affinity-purified rabbit anti-Corolla (animal 210) (1:2000) [76], mouse anti-C(3)G 1A8-1G2 (1:500) [77], anti-Cona (animal 20) (1:500) [78], high-affinity rat anti-HA (clone 3F10, Roche) (1:100), rabbit anti-histone H2AvD pS137 (1:500) (Rockland Inc.), mouse anti-γH2AV (1:1000) (Iowa Hybridoma Bank) [54], monoclonal mouse anti-GFP (1:500) (clone 3E6, Thermo Fisher Scientific) and rabbit anti-GFP (1:500) (AB6556, AbCam Inc.). Secondary goat anti-mouse, rabbit or rat Alexa-488, Alexa-555 and Alexa-647 IgG H&L chain conjugated antibodies were all used at 1:500 (Molecular Probes, Life Technologies, NY).

Images were acquired using a DeltaVision system (GE Healthcare) supplied with a 1x70 inverted microscope with a high-resolution CCD camera. Images were deconvolved using SoftWoRx v. 6.1 or 7.0.0 (Applied Precision/GE Healthcare) software. Image analysis was performed using either SoftWoRx v. 6.1 or Imaris software 8.3.1 (Bitplane, Zurich, Switzerland). Brightness and contrast were adjusted minimally to visualize signals during figure preparation.

## Supporting information

S1 Fig Narya, Nenya and Vilya are related to the Zip3-RNF212 family of homologs. (A) Protein alignment of *Drosophila melanogaster* Vilya (AAF45818), Narya (AAF48955) and Nenya (AAN14131). Proteins were aligned and visualized with MUSCLE and ClustalX programs using Jalview (http://www.jalview.org). Asterisks are shown above the conserved residues in the C3HC4 RING finger domain. The residues predicted to form a coiled-coil domain are below the black line. (B) A maximum-likelihood tree of the sequences from some members of both the Zip3-RNF212 group and the HEI10-like group, including *Caenorhabditis elegans* (Ce) ZHP-3 (NP_001250801), *Saccharomyces cerevisiae* (Sc) Zip3 (NP_013498), *Mus musculus* (Mm) RNF212 (F6TQD1) and HEI10 (NP_001104589), *Arabidopsis thaliana* (At) HEI10 (NP_175754), *Homo sapiens* (Hs) HEI10 (NP_878269), *Oryza sativa* (Os) HEI10 (EEE56612), *Zea mays* (Zm) HEI10 (NP_001152027), *Physcomitrella patens* (Pp) HEI10 (XP_001769363) and *Penicillium marneffei* (Pm) HEI10 (XP_002145282), and from *D*. *melanogaster* (Dm) Vilya (AAF45818), Narya (AAF48955) and Nenya (AAN14131) showing that all three *Drosophila* RING finger domain proteins cluster with the Zip3-RNF212 group. Similar results were previously reported for Vilya [16]. The maximum-likelihood tree was constructed using LG/G + I model with the MEGA 7 software (http://megasoftware.net) [80]. Scale bar indicates the number of nucleotide changes per site.
(TIF)

S2 Fig qPCR results of knockdown of *nenya* by RNAi. Relative quantities of *nenya* transcript done in triplicate in the listed genotypes. Error bars show ± SEM. *P = 0.03; n.s., statistically not significant, *P* = 0.78. Statistical test, two-sample t-test.
(TIF)

S3 Fig *narya* and *nenya* are required for DSB formation in both nurse cells and pro-oocytes. Maximum-intensity projection of deconvolved *z*-series through whole-mount germarium stained with DAPI and antibodies to C(3)G (red) to mark the SC and γH2AV (green) to mark the DSBs. In each panel, the tip of the germarium is pointed up. A schematic representation of a germarium is shown to the right. The dashed line indicates the location of the 16-cell early pachytene cysts (Region 2A), which is the developmental stage where programmed DNA DSBs are induced. The arrow indicates the one oocyte that has been selected in mid pachytene (Region 3). Endoreduplication cycles begin in region 3 in the supporting 15 nurse cells. The genotype of *narya* is *narya*^*+*^ (wildtype), *narya*^*JJ6*^ (null) or *narya*^*G4*^ (RING mutant) and all are in the absence of *nenya* using the RNAi transgene expressed with the *nosGAL4* driver. Scale, 5 μm.
(TIF)

S4 Fig No defect in DSB repair in *narya*^*G4*^ females in the absence of *nenya*. (A) Stage 2–3 egg chambers stained with DAPI (blue), C(3)G (red) to mark the oocyte and γH2AV (green) to mark the DSBs in the following genotypes: *narya*^*+*^ (*nosGAL4*/*+*; *nenya*^*RNAi*^/*+*), *narya*^*JJ6*^ (*nosGAL4 narya*^*JJ6*^/ *narya*^*JJ6*^; *nenya*^*RNAi*^/*+*) and *narya*^*G4*^ (*nosGAL4 narya*^*G4*^/ *narya*^*G4*^; *nenya*^*RNAi*^/*+*). No DSBs are found in the oocyte nucleus (dashed box), which would indicate a delay in DSB repair. DSBs within the 15 nurse cells are from endoreduplication cycles. (B) Karyosome stained with DAPI from a Stage 8 egg chamber showing that the structure of the karyosome is not fragmented in the absence of *nenya* (*nosGAL4*/*+*; *nenya*^*RNAi*^/*+*), in the double mutant (*nosGAL4 narya*^*JJ6*^/ *narya*^*JJ6*^; *nenya*^*RNAi*^/*+*), or in the *narya*^*G4*^ double mutant (*nosGAL4 narya*^*G4*^/ *narya*^*G4*^; *nenya*^*RNAi*^/*+*) where DSBs are formed but not repaired into crossovers. For each genotype, 100% of the karyosomes were shaped normally (n = 5). Arrowhead indicates the karyosome. Scale, 5 μm.
(TIF)

S5 Fig *narya*^*GFPcrispr*^ expression within the germarium. Two examples of germaria expressing *narya*^*GFPcrispr*^ showing both the undeconvolved and deconvolved images for each. *narya*^*GFPcrispr*^ expression can be seen in the undeconvolved images as a haze in early pachytene nuclei (Region 2A, dashed line), as well as discrete foci that begin in early pachytene and persist in pro-oocytes as the cysts progress. The primary Narya^GFP^ staining in the deconvolved images is the discrete foci that persist throughout pachytene as the cysts develop. Images are maximum-intensity projections of *z*-series through the entire germarium stained with DAPI (blue), Corolla (red) to mark the SC and GFP (green) to mark Narya. Scale, 5 μm.
(TIF)

S6 Fig Gallery of *z*-slices showing the colocalization of Narya with DSB sites. An early pachytene (Region 2A) pro-oocyte of the genotype *narya*^*GFPcrispr*^ stained with antibodies to Cona (blue) to mark the pro-oocytes, γH2AV (red) to mark the DSBs and GFP (green) to mark Narya. Images are single *z*-slices of 0.2 μm throughout the SC of the nucleus. Scale, 1 μm.
(TIF)

S7 Fig Gallery of *z*-slices showing the colocalization of Narya with Vilya. Two sets of serial *z*-slices of early pachytene (Region 2A) pro-oocytes of the genotype *narya*^*GFPcrispr*^/*nosGAL4 narya*^*GFPcrispr*^; *PUASp-vilya*^*3XHA*^*/+* stained with antibodies to Corolla (blue) to mark the pro-oocytes, HA (red) to mark Vilya and GFP (green) to mark Narya. Images are single *z*-slices of 0.2 μm thickness throughout the SC region of the nucleus. The presence of the Narya foci prior to the Vilya foci in the *z*-series is an artifact of resolution in *z* not being perfect. Scale, 1 μm.
(TIF)

S8 Fig Narya and Nenya’s interaction with Vilya is not dependent on Vilya’s RING finger domain or the C-terminal residues of Vilya. (A) Vilya’s RING finger domain is not required for its interaction with Narya in a yeast two-hybrid assay. Each of the conserved cysteines and the histidine in the RING finger domain were mutated individually to either a serine (for the cysteines) or a tyrosine (for the histidine). (B) Similarly, the RING finger domain of Vilya is also not required for its interaction with Nenya. The RING finger domain of Vilya is required for Vilya’s interaction with MEI-P22 [16]. (C) The truncation mutant Vilya^826^ that deletes the C-terminal 24 residues of Vilya and is known to cause segregation errors in the fly is still able to interact with Narya and Nenya by yeast two-hybrid. In each experiment, six twofold dilutions of equal starting amounts were plated on each of the selection plates.
(TIF)

S9 Fig Unlike Vilya, Narya and Nenya do not interact with MEI-P22. Vilya is the only one of the three RING finger proteins required for meiotic DSB formation that interacts with MEI-P22 by yeast two-hybrid assay [16]. Control plasmids were supplied by Clontech (pGBKT7-53 and pGADT7-T). In each experiment, six twofold dilutions of equal starting amounts were plated on each of the selection plates.
(TIF)

S10 Fig Lack of yeast two-hybrid interaction of Narya^G4^ with RING finger domain-containing proteins is not due to lack of expression. Western blot analysis showing that Narya^G4^ is expressed in the Y187 strain carrying *pGBKT7-narya*^*G4*^ and the AH109 strain carrying *pGADT7-narya*^*G4*^. GAL4-BD-cMyc (empty vector) is predicted to be 22 kDa and the GAL4-AD-HA (empty vector) is predicted to be 24kDa, making each of the Narya^G4^ fusions 43 and 45 kDa in size, respectively.
(TIF)

S1 Table Tajima's relative rate tests for *narya* and *nenya*. *narya* and *nenya* sequence from each species listed was compared to the *nenya* sequence in *D*. *pseduoobscura* (the most common ancestor analyzed prior to the gene duplication event) using the Tajima’s relative rate test. The *P* values indicate there is no significant difference in the rate of divergence between *narya* and *nenya*.
(DOCX)

S2 Table Nondisjunction rates in mutants that fail to form crossovers due to the absence of either DSBs or SC. Published *X* chromosome nondisjunction rates for DSB-defective mutants (*mei-W68* [2],*mei-P22*^*103*^ [14], *trem*^*F9*^ [15] and *vilya*^*826*^ [16]) and SC-defective mutants (*c3g*^*68*^ [81], *cona*^*A12*^ [78] and *corolla*^*1*^ [76]).
(DOCX)

S3 Table *narya*^*GFPcrispr*^ is a fully functional allele. Females of the above genotype were crossed to *y sc cv v f·car* / *B*^*s*^*Y* males. This cross allows for the identification of normal offspring (*XX* females, *XY* males), diplo-*X* and nullo-*X* exceptions. The table shows the summed nondisjunction frequency (% *X* ND). The total number of progeny scored are adjusted to account for the inviable progeny class (Adj total, see Methods).
(DOCX)
