# Supplementary figures and images for: Narya, a RING finger domain-containing protein, is required for meiotic DNA double-strand break formation and crossover maturation in Drosophila melanogaster
Source: PLoS Genet. 2019 Jan 7;15(1):e1007886. doi: 10.1371/journal.pgen.1007886 (PMC6336347; doi:10.1371/journal.pgen.1007886)

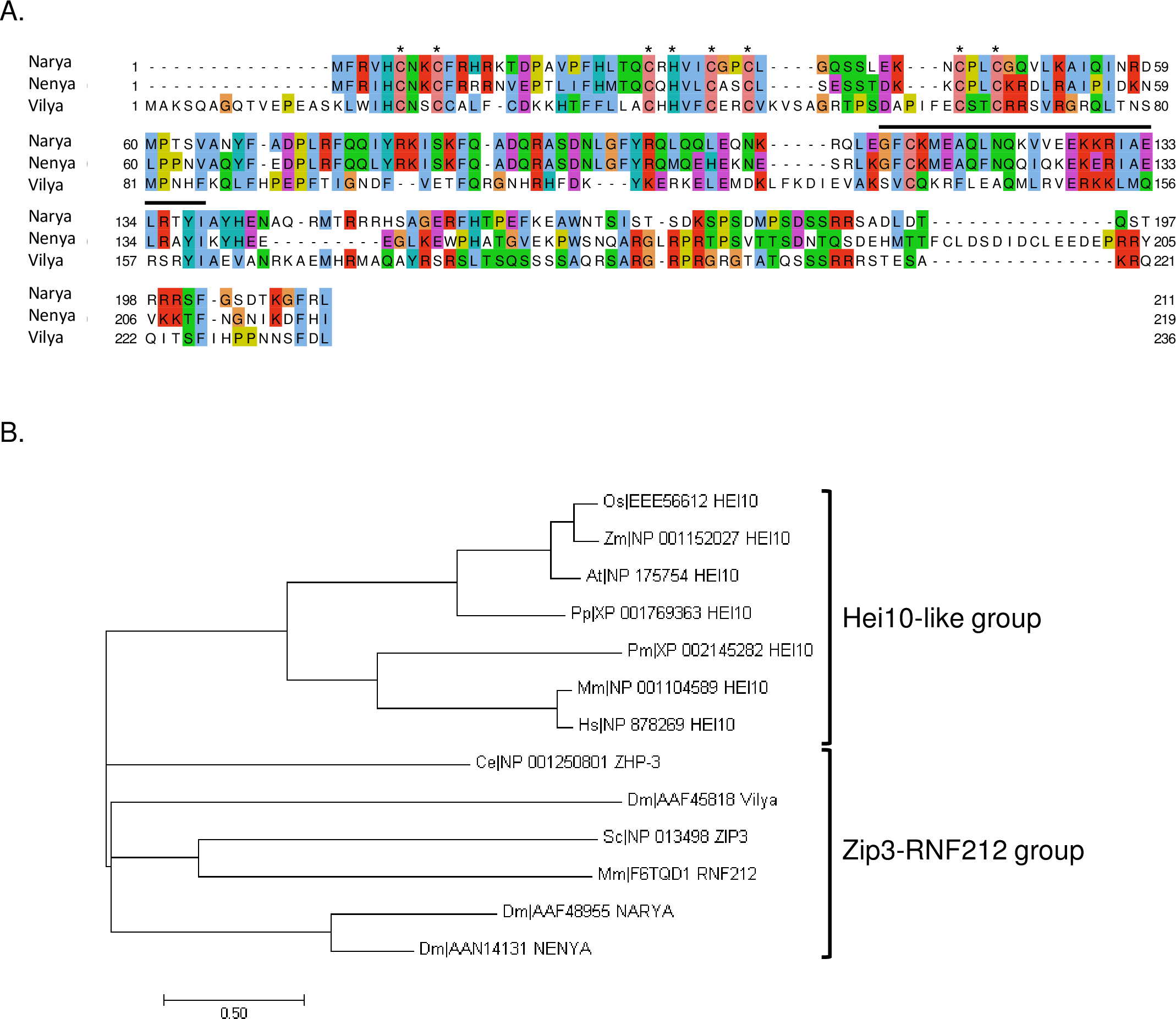

Supplement: S1 Fig — (A) Protein alignment of Drosophila melanogaster Vilya (AAF45818), Narya (AAF48955) and Nenya (AAN14131). Proteins were aligned and visualized with MUSCLE and ClustalX programs using Jalview (http://www.jalview.org). Asterisks are shown above the conserved residues in the C3HC4 RING finger domain. The residues predicted to form a coiled-coil domain are below the black line. (B) A maximum-likelihood tree of the sequences from some members of both the Zip3-RNF212 group and the HEI10-like group, including Caenorhabditis elegans (Ce) ZHP-3 (NP_001250801), Saccharomyces cerevisiae (Sc) Zip3 (NP_013498), Mus musculus (Mm) RNF212 (F6TQD1) and HEI10 (NP_001104589), Arabidopsis thaliana (At) HEI10 (NP_175754), Homo sapiens (Hs) HEI10 (NP_878269), Oryza sativa (Os) HEI10 (EEE56612), Zea mays (Zm) HEI10 (NP_001152027), Physcomitrella patens (Pp) HEI10 (XP_001769363) and Penicillium marneffei (Pm) HEI10 (XP_002145282), and from D. melanogaster (Dm) Vilya (AAF45818), Narya (AAF48955) and Nenya (AAN14131) showing that all three Drosophila RING finger domain proteins cluster with the Zip3-RNF212 group. Similar results were previously reported for Vilya [16]. The maximum-likelihood tree was constructed using LG/G + I model with the MEGA 7 software (http://megasoftware.net) [80]. Scale bar indicates the number of nucleotide changes per site. (TIF) [file pgen.1007886.s001.tif]

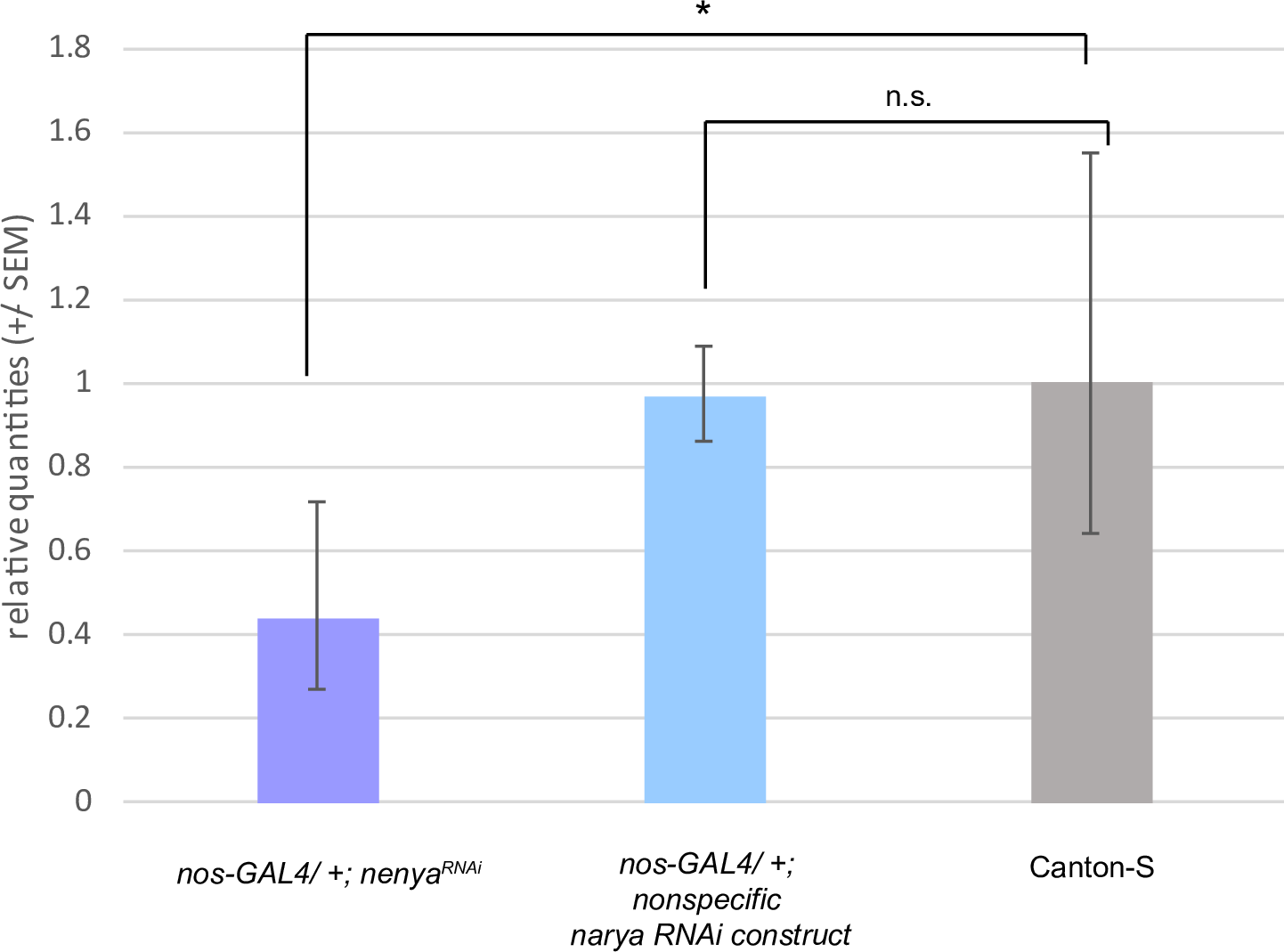

Supplement: S2 Fig — Relative quantities of nenya transcript done in triplicate in the listed genotypes. Error bars show ± SEM. *P = 0.03; n.s., statistically not significant, P = 0.78. Statistical test, two-sample t-test. (TIF) [file pgen.1007886.s002.tif]

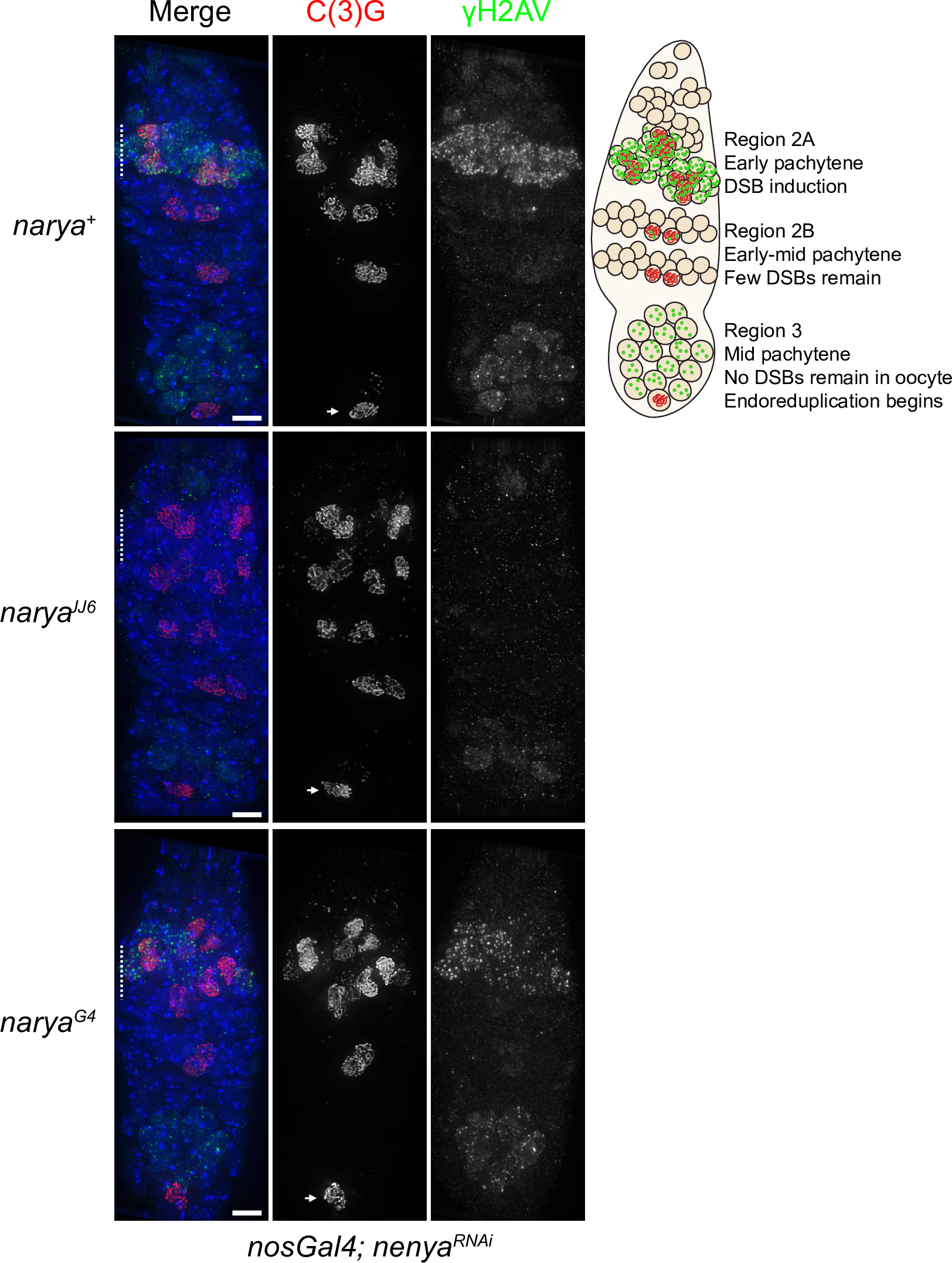

Supplement: S3 Fig — Maximum-intensity projection of deconvolved z-series through whole-mount germarium stained with DAPI and antibodies to C(3)G (red) to mark the SC and γH2AV (green) to mark the DSBs. In each panel, the tip of the germarium is pointed up. A schematic representation of a germarium is shown to the right. The dashed line indicates the location of the 16-cell early pachytene cysts (Region 2A), which is the developmental stage where programmed DNA DSBs are induced. The arrow indicates the one oocyte that has been selected in mid pachytene (Region 3). Endoreduplication cycles begin in region 3 in the supporting 15 nurse cells. The genotype of narya is narya+ (wildtype), naryaJJ6 (null) or naryaG4 (RING mutant) and all are in the absence of nenya using the RNAi transgene expressed with the nosGAL4 driver. Scale, 5 μm. (TIF) [file pgen.1007886.s003.tif]

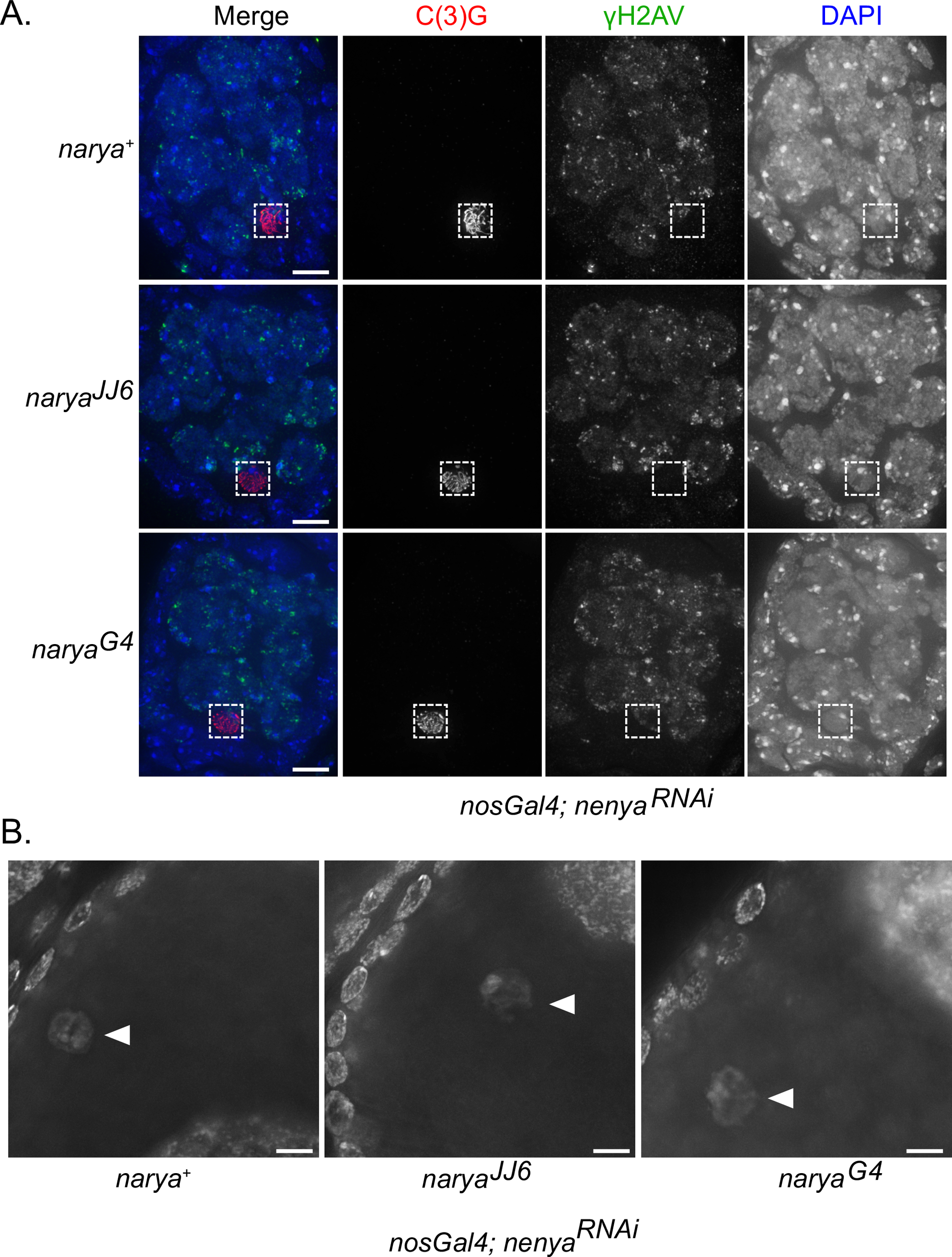

Supplement: S4 Fig — (A) Stage 2–3 egg chambers stained with DAPI (blue), C(3)G (red) to mark the oocyte and γH2AV (green) to mark the DSBs in the following genotypes: narya+ (nosGAL4/+; nenyaRNAi/+), naryaJJ6 (nosGAL4 naryaJJ6/ naryaJJ6; nenyaRNAi/+) and naryaG4 (nosGAL4 naryaG4/ naryaG4; nenyaRNAi/+). No DSBs are found in the oocyte nucleus (dashed box), which would indicate a delay in DSB repair. DSBs within the 15 nurse cells are from endoreduplication cycles. (B) Karyosome stained with DAPI from a Stage 8 egg chamber showing that the structure of the karyosome is not fragmented in the absence of nenya (nosGAL4/+; nenyaRNAi/+), in the double mutant (nosGAL4 naryaJJ6/ naryaJJ6; nenyaRNAi/+), or in the naryaG4 double mutant (nosGAL4 naryaG4/ naryaG4; nenyaRNAi/+) where DSBs are formed but not repaired into crossovers. For each genotype, 100% of the karyosomes were shaped normally (n = 5). Arrowhead indicates the karyosome. Scale, 5 μm. (TIF) [file pgen.1007886.s004.tif]

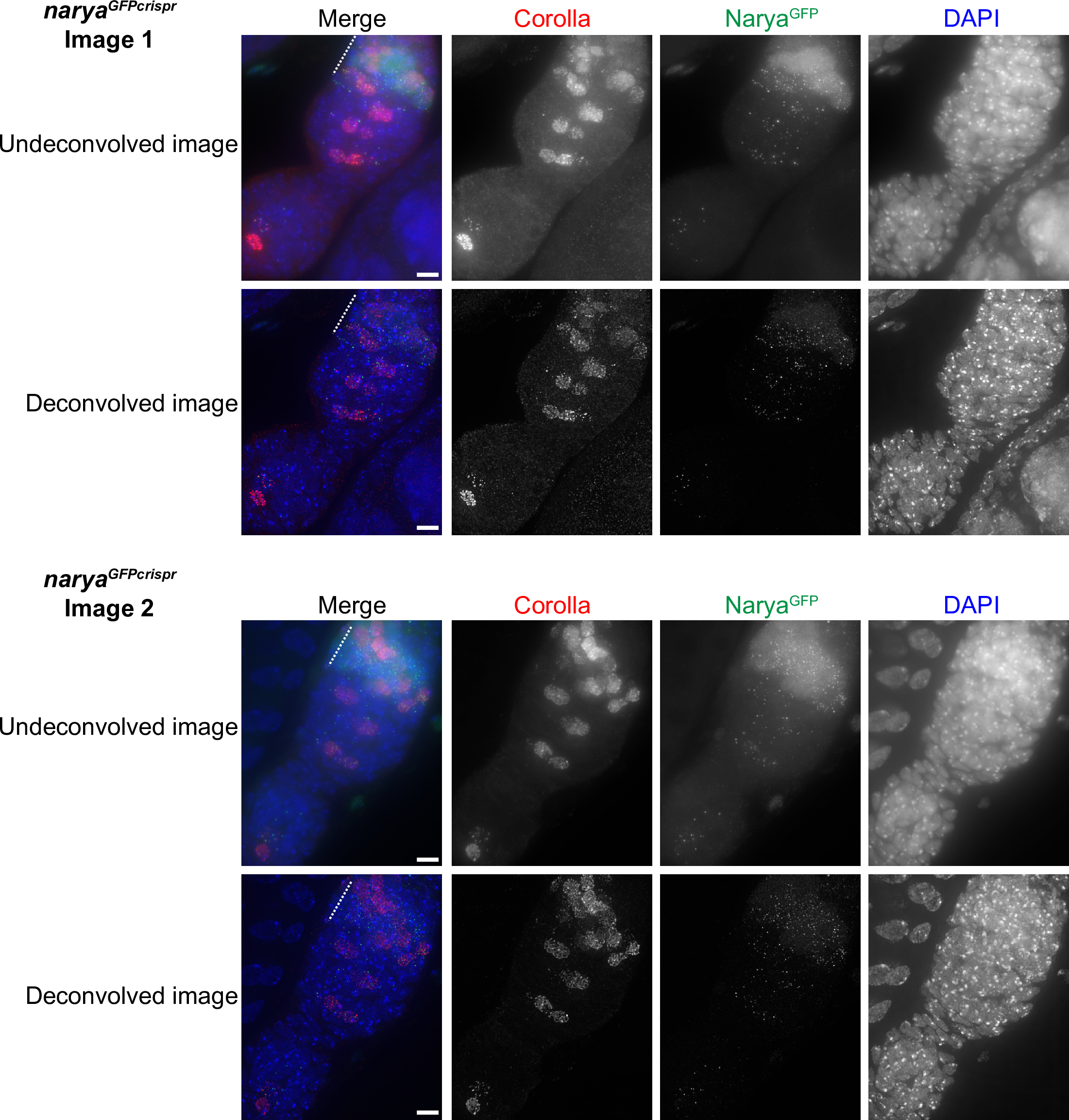

Supplement: S5 Fig — Two examples of germaria expressing naryaGFPcrispr showing both the undeconvolved and deconvolved images for each. naryaGFPcrispr expression can be seen in the undeconvolved images as a haze in early pachytene nuclei (Region 2A, dashed line), as well as discrete foci that begin in early pachytene and persist in pro-oocytes as the cysts progress. The primary NaryaGFP staining in the deconvolved images is the discrete foci that persist throughout pachytene as the cysts develop. Images are maximum-intensity projections of z-series through the entire germarium stained with DAPI (blue), Corolla (red) to mark the SC and GFP (green) to mark Narya. Scale, 5 μm. (TIF) [file pgen.1007886.s005.tif]

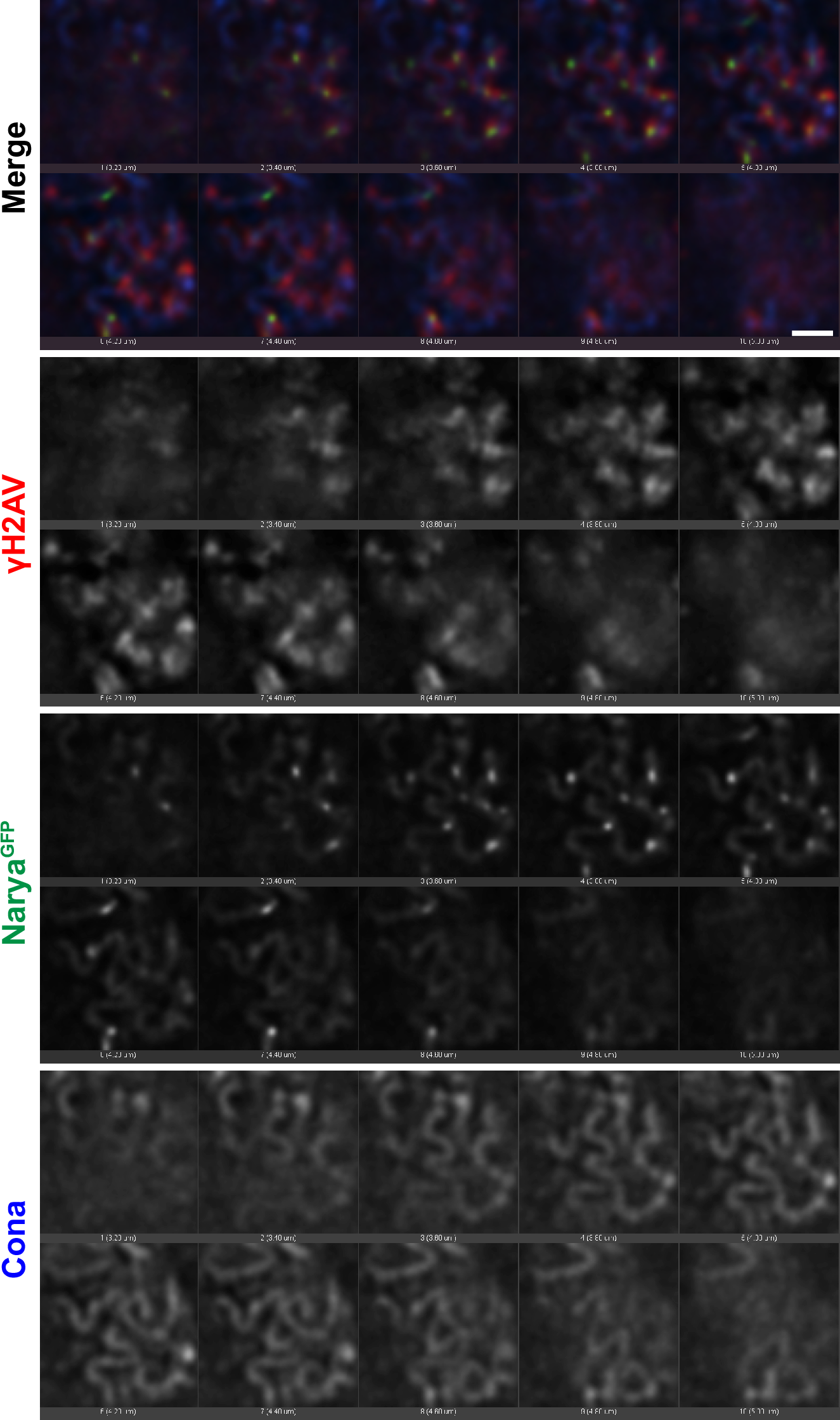

Supplement: S6 Fig — An early pachytene (Region 2A) pro-oocyte of the genotype naryaGFPcrispr stained with antibodies to Cona (blue) to mark the pro-oocytes, γH2AV (red) to mark the DSBs and GFP (green) to mark Narya. Images are single z-slices of 0.2 μm throughout the SC of the nucleus. Scale, 1 μm. (TIF) [file pgen.1007886.s006.tif]

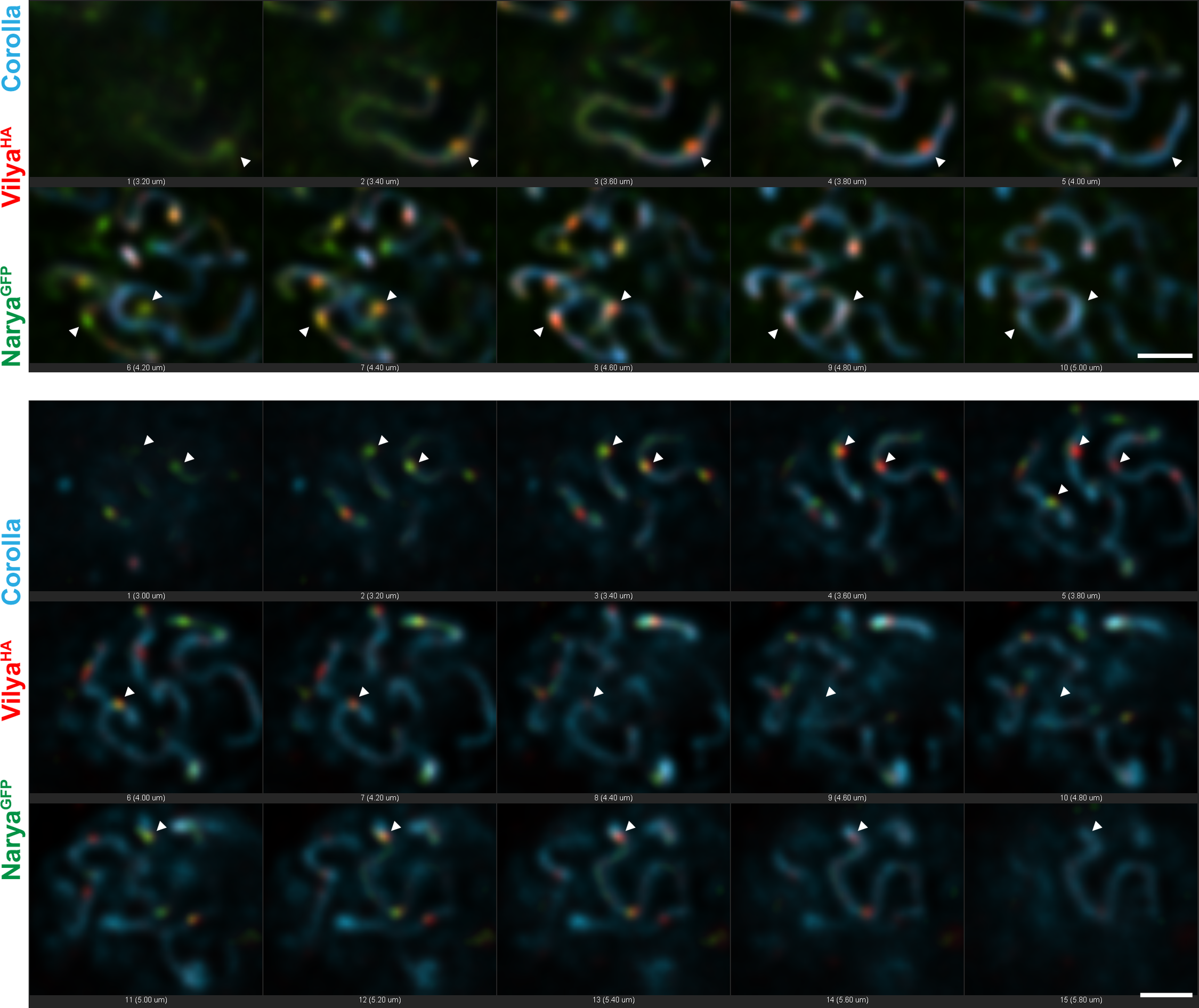

Supplement: S7 Fig — Two sets of serial z-slices of early pachytene (Region 2A) pro-oocytes of the genotype naryaGFPcrispr/nosGAL4 naryaGFPcrispr; PUASp-vilya3XHA/+ stained with antibodies to Corolla (blue) to mark the pro-oocytes, HA (red) to mark Vilya and GFP (green) to mark Narya. Images are single z-slices of 0.2 μm thickness throughout the SC region of the nucleus. The presence of the Narya foci prior to the Vilya foci in the z-series is an artifact of resolution in z not being perfect. Scale, 1 μm. (TIF) [file pgen.1007886.s007.tif]

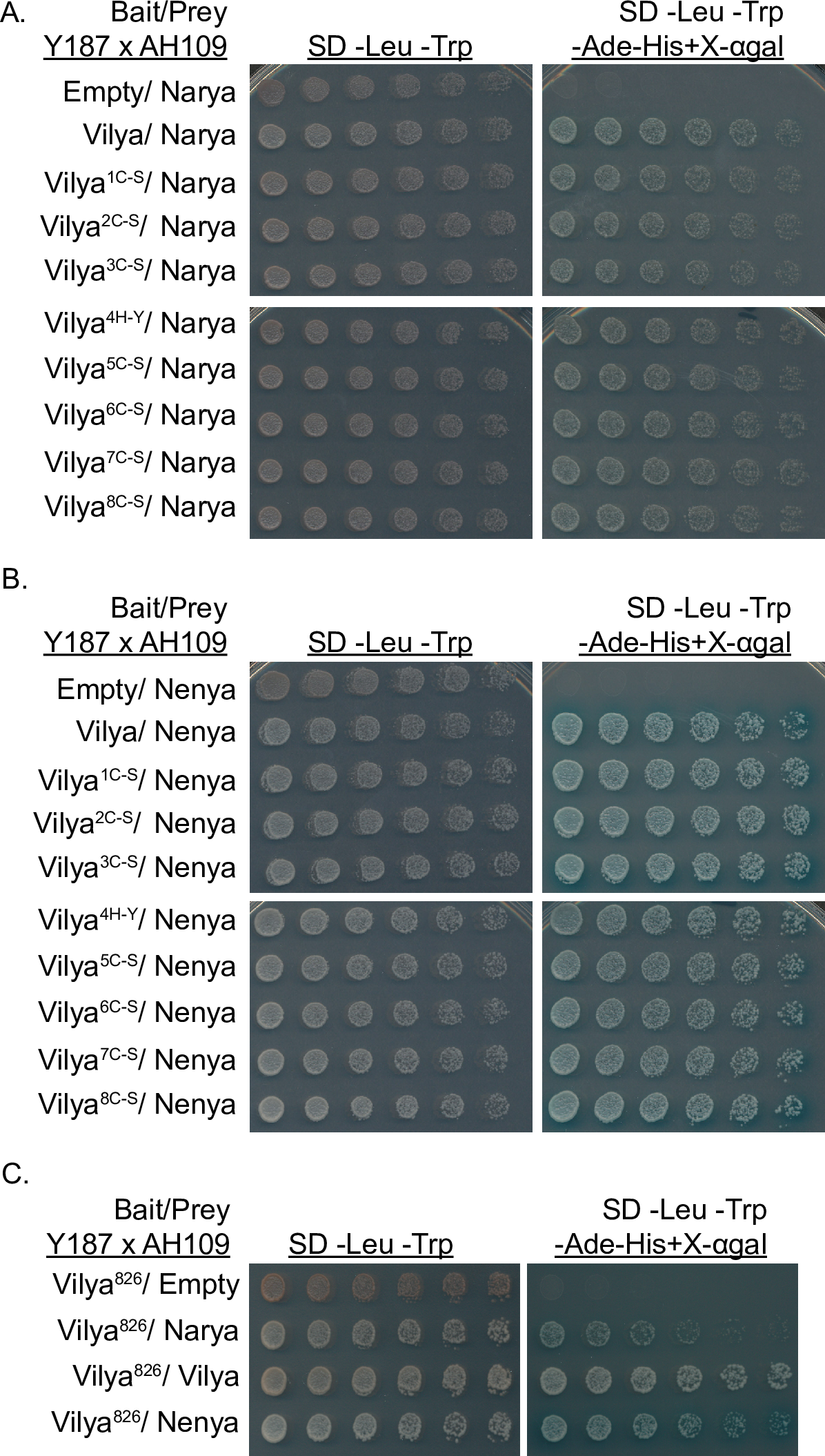

Supplement: S8 Fig — (A) Vilya’s RING finger domain is not required for its interaction with Narya in a yeast two-hybrid assay. Each of the conserved cysteines and the histidine in the RING finger domain were mutated individually to either a serine (for the cysteines) or a tyrosine (for the histidine). (B) Similarly, the RING finger domain of Vilya is also not required for its interaction with Nenya. The RING finger domain of Vilya is required for Vilya’s interaction with MEI-P22 [16]. (C) The truncation mutant Vilya826 that deletes the C-terminal 24 residues of Vilya and is known to cause segregation errors in the fly is still able to interact with Narya and Nenya by yeast two-hybrid. In each experiment, six twofold dilutions of equal starting amounts were plated on each of the selection plates. (TIF) [file pgen.1007886.s008.tif]

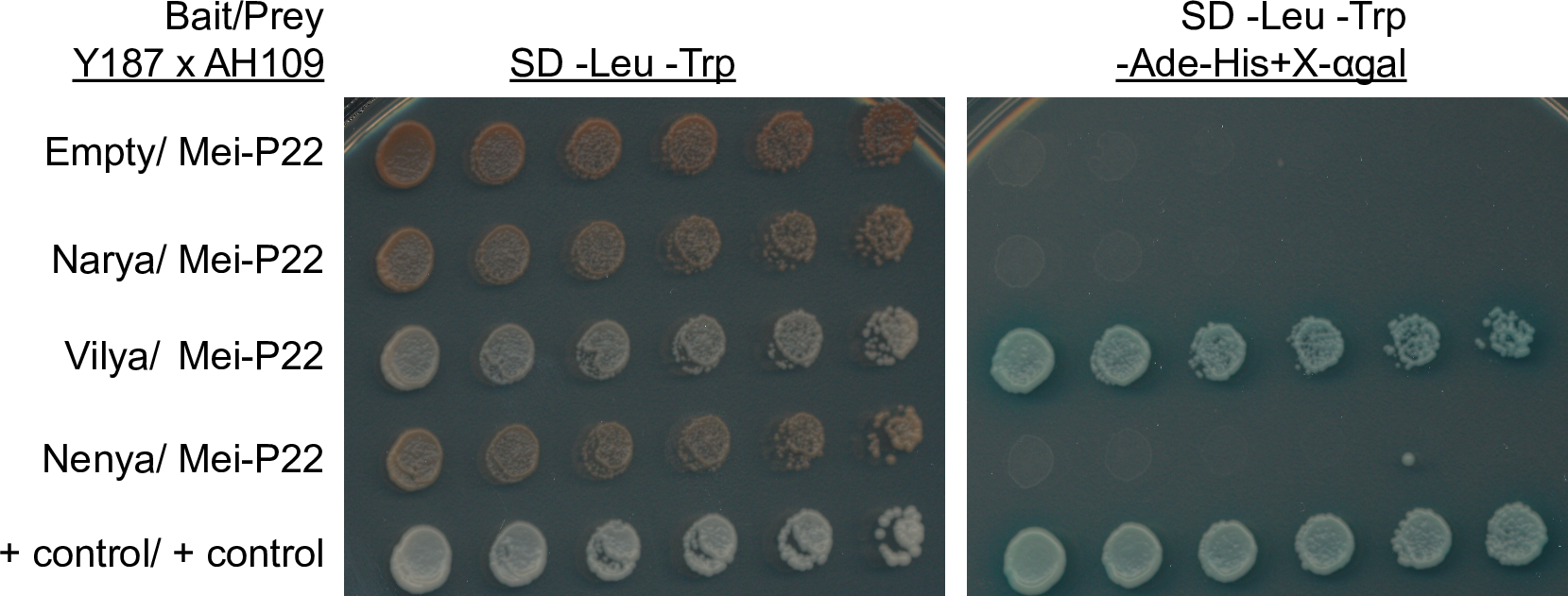

Supplement: S9 Fig — Vilya is the only one of the three RING finger proteins required for meiotic DSB formation that interacts with MEI-P22 by yeast two-hybrid assay [16]. Control plasmids were supplied by Clontech (pGBKT7-53 and pGADT7-T). In each experiment, six twofold dilutions of equal starting amounts were plated on each of the selection plates. (TIF) [file pgen.1007886.s009.tif]

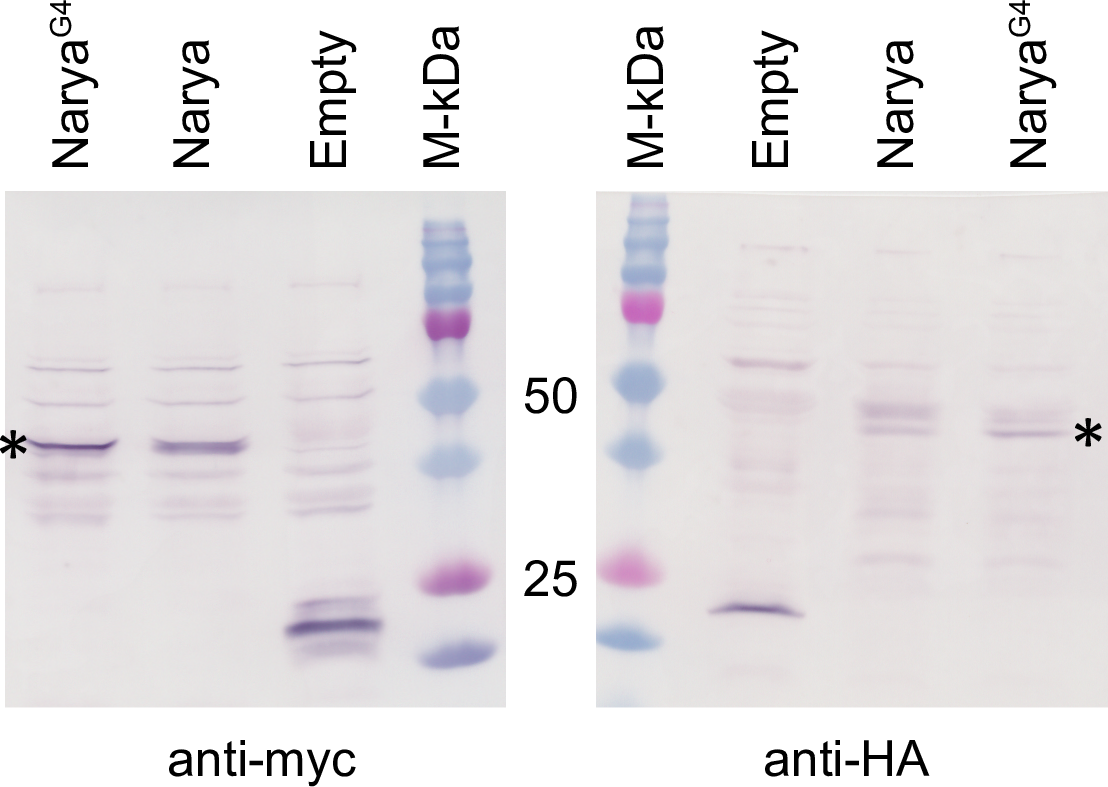

Supplement: S10 Fig — Western blot analysis showing that NaryaG4 is expressed in the Y187 strain carrying pGBKT7-naryaG4 and the AH109 strain carrying pGADT7-naryaG4. GAL4-BD-cMyc (empty vector) is predicted to be 22 kDa and the GAL4-AD-HA (empty vector) is predicted to be 24kDa, making each of the NaryaG4 fusions 43 and 45 kDa in size, respectively. (TIF) [file pgen.1007886.s010.tif]
