# Supplementary material for: Narya, a RING finger domain-containing protein, is required for meiotic DNA double-strand break formation and crossover maturation in Drosophila melanogaster
Source: PLoS Genet. 2019 Jan 7;15(1):e1007886. doi: 10.1371/journal.pgen.1007886 (PMC6336347; doi:10.1371/journal.pgen.1007886)
Supplement: S1 Table — narya and nenya sequence from each species listed was compared to the nenya sequence in D. pseduoobscura (the most common ancestor analyzed prior to the gene duplication event) using the Tajima’s relative rate test. The P values indicate there is no significant difference in the rate of divergence between narya and nenya. (DOCX) [file pgen.1007886.s011.docx]

| **Species** | **Outgroup species** | ***P* value^a^** |
| --- | --- | --- |
| *D. melanogaster* | *D. pseudoobscura* | 0.14 |
| *D. simulans* | *D. pseudoobscura* | 0.61 |
| *D. sechellia* | *D. pseudoobscura* | 0.39 |
| *D. erecta* | *D. pseudoobscura* | 0.03 |
| *D. yakuba* | *D. pseudoobscura* | 0.01 |

^a^ significance indicates different mutation rates
