# Supplementary material for: Narya, a RING finger domain-containing protein, is required for meiotic DNA double-strand break formation and crossover maturation in Drosophila melanogaster
Source: PLoS Genet. 2019 Jan 7;15(1):e1007886. doi: 10.1371/journal.pgen.1007886 (PMC6336347; doi:10.1371/journal.pgen.1007886)
Supplement: S2 Table — Published X chromosome nondisjunction rates for DSB-defective mutants (mei-W68 [2],mei-P22103 [14], tremF9 [15] and vilya826 [16]) and SC-defective mutants (c3g68 [81], conaA12 [78] and corolla1 [76]). (DOCX) [file pgen.1007886.s012.docx]

| **Genotype** | **Meiotic process affected**  **by mutant** | **% *X* ND^a^** |
| --- | --- | --- |
| *mei-W68* | DSB formation | 38.7 |
| *mei-P22^103^* | DSB formation | 37.4 |
| *trem^F9^* | DSB formation | 33.4 |
| *vilya^826^* | DSB formation | 41.1 |
| *c3g^68^* | SC formation | 39.2 |
| *cona^A12^* | SC formation | 36.5 |
| *corolla^1^* | SC formation | 44.5 |

^a^ ND, nondisjunction
