# Supplementary material for: Narya, a RING finger domain-containing protein, is required for meiotic DNA double-strand break formation and crossover maturation in Drosophila melanogaster
Source: PLoS Genet. 2019 Jan 7;15(1):e1007886. doi: 10.1371/journal.pgen.1007886 (PMC6336347; doi:10.1371/journal.pgen.1007886)
Supplement: S3 Table — Females of the above genotype were crossed to y sc cv v f·car / BsY males. This cross allows for the identification of normal offspring (XX females, XY males), diplo-X and nullo-X exceptions. The table shows the summed nondisjunction frequency (% X ND). The total number of progeny scored are adjusted to account for the inviable progeny class (Adj total, see Methods). (DOCX) [file pgen.1007886.s013.docx]

| **Genotype** | **Copies of *narya^GFPcrispr^* gene** | **Functional copies of *nenya* gene** | **Knockdown of *nenya* by RNAi** | **% *X* ND^a^** | **Adj total progeny scored** |
| --- | --- | --- | --- | --- | --- |
| *narya^GFPcrispr^/narya^GFPcrispr^* | +/+ | +/+ | — | 0.3 | 669 |
| *nosGAL4 narya^GFPcrispr^/narya^GFPcrispr^* | +/+ | +/+ | — | 0.6 | 668 |
| *narya^GFPcrispr^/narya^GFPcrispr^; nenya^del^/+* | +/+ | +/- | — | 0.0 | 1003 |
| *narya^GFPcrispr^/narya^GFPcrispr^; nenya^del^/nenya^del^* | +/+ | -/- | — | 1.0 | 407 |
| *nosGAL4 narya^GFPcrispr^/narya^GFPcrispr^; nenya^RNAi^/+* | +/+ | +/+ | yes | 0.7 | 1132 |

^a^ ND, nondisjunction
